# Block Copolymers: Synthesis, Self-Assembly, and Applications

**DOI:** 10.3390/polym9100494

**Published:** 2017-10-09

**Authors:** Hongbo Feng, Xinyi Lu, Weiyu Wang, Nam-Goo Kang, Jimmy W. Mays

**Affiliations:** 1 Department of Chemistry, University of Tennessee, Knoxville, TN 37996, USA; hfeng9@utk.edu (H.F.); xlu8@utk.edu (X.L.); nkang1@utk.edu (N.-G.K.); 2 Center for Nanophase Materials Sciences, Oak Ridge National Laboratory, Oak Ridge, TN 37830, USA; wwang41@utk.edu; 3 Chemical Sciences Division, Oak Ridge National Laboratory, Oak Ridge, TN 37830, USA

**Keywords:** block copolymers, synthesis, self-assembly, applications

## Abstract

Research on block copolymers (BCPs) has played a critical role in the development of polymer chemistry, with numerous pivotal contributions that have advanced our ability to prepare, characterize, theoretically model, and technologically exploit this class of materials in a myriad of ways in the fields of chemistry, physics, material sciences, and biological and medical sciences. The breathtaking progress has been driven by the advancement in experimental techniques enabling the synthesis and characterization of a wide range of block copolymers with tailored composition, architectures, and properties. In this review, we briefly discussed the recent progress in BCP synthesis, followed by a discussion of the fundamentals of self-assembly of BCPs along with their applications.

## 1. Introduction

**Figure 1:**
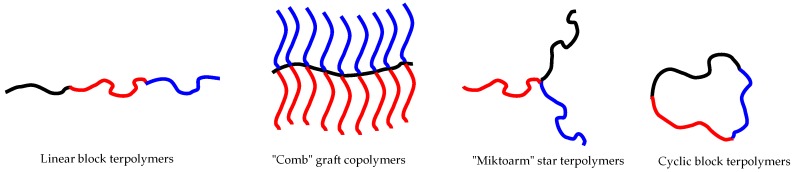
Representative architectures of linear block terpolymers, “comb” graft polymers, miktoarm star terpolymers, and cyclic block terpolymers.

Block copolymers (BCPs) are a specific class of copolymers, in which the chemically distinct monomer units are grouped in discrete blocks along the polymer chain [1]. Figure 1 illustrates a few of the many architectures of BCPs, which can be configured into linear, branched (graft and star), and cyclic molecular architectures. Thanks to the advancement of polymer synthetic strategies and techniques, e.g., controlled polymerization techniques along with facile post-polymerization functionalization, BCPs with precisely controlled molecular weights and defined macromolecular architectures can be prepared [2,3,4,5,6,7,8]. The extraordinary structural and compositional versatility of BCPs has facilitated an explosion in the discovery and implementation of innovative synthetic strategies capable of generating previously unattainable levels of man-made architectural complexity.

**Figure 2:**
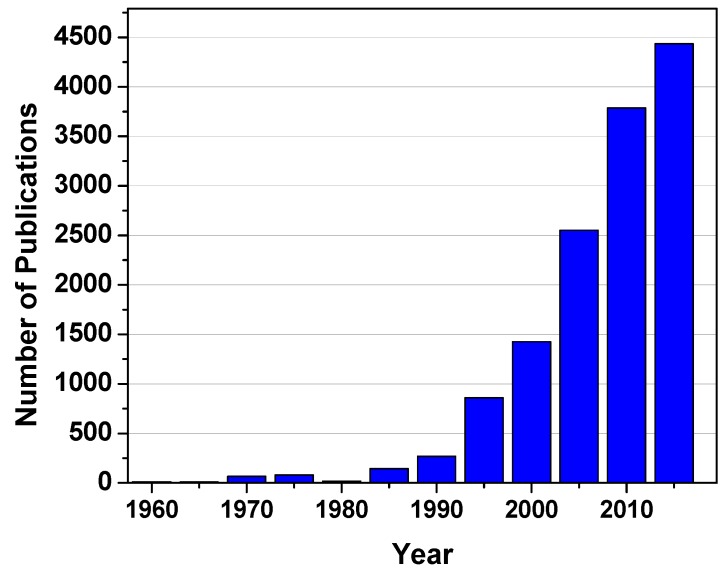
The number of publications with block copolymer as topic against year. The data were obtained from Web of Science (2017 Clarivate Analytics).

One of the ubiquitous features of BCPs is their ability to form a plethora of nanoscale ordered structures. By manipulating the molecular parameters such as the Flory-Huggins interaction parameter (χ), the degree of polymerization (N), and the volume fraction (*f*), various morphologies including spherical, cylindrical, lamellar, and others have been revealed both experimentally and theoretically [9,10]. Furthermore, recent studies have demonstrated that the macromolecular architecture is another key factor in controlling both the resulting morphologies and their extent of long range order [11]. Due to these fascinating features, research on BCPs has long been a popular topic worldwide. Figure 2 shows the number of publications with the topic “block copolymer” over the past five decades. 

In addition to the academic interest in BCPs, the scope of applications for BCPs has been rapidly expanding to the fields of advanced materials (e.g., thermoplastic elastomers), drug delivery, patterning, porous materials, and many others over the last two decades [12]. Thermoplastic elastomers take advantage of the combination of rubbery segments and rigid segments within block copolymers. Drug encapsulation and delivery are facilitated by the amphiphilicity of block copolymers in solution. The application of block copolymers in both the soft lithography and synthesis of porous materials is based on the various nanoscale morphologies induced by self-assembly. Each of the applications mentioned above will be elaborated on in the main text. 

This review briefly covers the recent breathtaking progress in the synthesis, self-assembly behavior, and applications of BCPs. Section 2 briefly describes the synthetic strategies for the preparation of BCPs. Section 3 discusses BCP self-assembly in bulk and in solution. Section 4 presents several major applications of BCPs in thermoplastic elastomer, drug delivery, soft lithography, and porous material applications. The conclusions and future perspectives of BCP research are presented in the last section. We hope that this review will attract emerging researchers to this field and advance the understanding and utilization of the fascinating BCP systems. 

## 2. Synthesis of BCPs

Significant work has been focused on the synthesis of BCPs with structural and composition variety. The synthetic strategies mainly include: (1) the sequential addition of monomers via “living”/controlled polymerization techniques; and (2) coupling reactions exploiting the active chain-ends of different chain segments [13].

### 2.1. Sequential Addtion Polymerization

#### 2.1.1. Controlled Polymerization

Controlled radical polymerization (CRP) techniques represent the most versatile and facile approach for BCP synthesis, mainly due to their compatibility with a wide spectrum of monomers, high tolerance of functional groups and impurities, and ease of experimental setup [14,15,16]. The preparation of BCPs using sequential CRP relies on the fact that the reactive polymer chain end is preserved by a reversible reaction between the active species and the dormant species [17,18,19]. Over the last two decades, numerous literature has described the synthesis of BCPs via controlled polymerization techniques including: atom transfer racial polymerization (ATRP), reversible addition-fragmentation chain transfer (RAFT) radical polymerization, nitroxide-mediated polymerization (NMP), and many others [20,21,22]. 

**Scheme 1:**
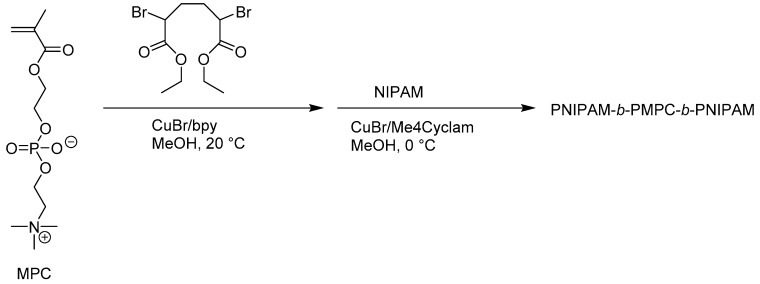
Synthesis of the poly(*N*-isopropyl acrylamide-*b*-2-methacryloyloxyethylphosphorylcholine-*b*-*N*-isopropyl acrylamide) (PNIPAM-*b*-PMPC-*b*-PNIPAM) triblock copolymers via atom transfer racial polymerization (ATRP) using a bifunctional ATRP initiator.

Polymer chain ends equipped with an alkyl halide enables ATRP to prepare BCPs through the addition of a second monomer. For example, a series of biocompatible, thermo-responsive ABA triblock copolymers comprising poly(*N*-isopropyl acrylamide) (PNIPAM) as the A block and poly(2-methacryloyloxyethyl phosphorylcholine) (PMPC) as the central B block was synthesized using ATRP initiated by a difunctional initiator, as illustrated in Scheme 1 [23]. An α,ω-Br terminated PMPC was first synthesized in methanol, purified, and used subsequently for the polymerization of NIPAM. This doubly amphiphilic BCP exhibits interesting thermosensitive properties, whereas above the lower critical solution temperature (LCST) of PNIPAM, the BCP self-assembles into a physical gel. The phosphoryl choline moiety is an important component of cell membranes. The cell viability experiments confirmed that these thermo-responsive gels are sufficiently biocompatible to act as a culture medium for hamster lung cells.

**Scheme 2:**
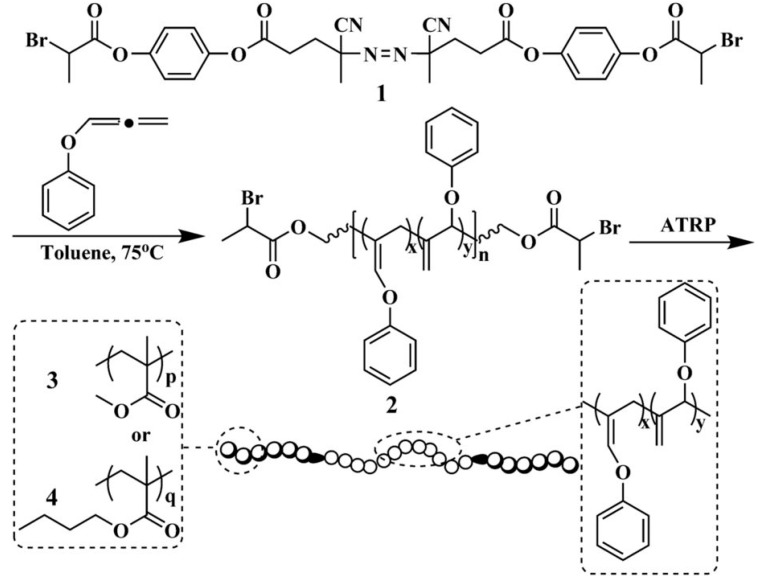
Synthesis of polyallene-based triblock copolymer using conventional free radical and ATRP. Reprinted from Reference [30]. (Copyright (2017) Nature Publishing Group).

Matyjaszewski et al. reported the preparation of poly(*n*-butyl acrylate-*b*-methyl methacrylate) (PBA-*b*-PMMA) by activators regenerated by electron transfer atom transfer radical polymerization (ARGET ATRP) with ppm levels of Cu catalyst [24]. Controlled polymerization was realized using tris(2-pyridylmethyl)amine (TPMA) as a ligand because of its strong binding interaction to copper. This new ARGET system was also successfully applied to the efficient synthesis of styrene and *n*-butyl acrylate block copolymers [25]. Later on, miniemulsion ARGET ATRP was developed by the same group and used for the preparation of homopolymers and block copolymers [26,27]. However, ATRP still does not work for some monomers, though it has many advantages compared to other polymerization methods for block copolymerization. For example, some monomers contain functionality that can form complexes with transition metal catalyst and have a detrimental effect on polymerization, such as side reactions resulting in a broad polydispersity index (PDI) [15]. Tremendous efforts have been devoted to developing novel ligands to minimize such side reactions [28]. ATRP-based dispersion polymerizations have also been developed. Armes and coworkers synthesized zwitterionic PMPC-based block copolymers by a dispersion ATRP process [29]. PEG–Br was used as macroinitiator with CuBr/ 2,2′-bipyridine as the catalyst for the dispersion polymerization of PMPC in isopropanol/water (9:1, *w*/*w*). Using ethylene glycol dimethacrylate (EGDMA) as a crosslinker, hydrogel particles with controllable sizes were obtained. The particle size could be controlled by the block composition and initial MPC concentration. Using a similar strategy, the same group synthesized well-defined PEG-*b*-PDMAEMA-*b*-PMPC. Huang and coworkers synthesized a series of ABA triblock copolymers, consisting of double-bond-containing poly(phenoxyallene) (PPOA), PMMA, or PBA segments using sequential free radical polymerization and ATRP [30]. A bifunctional initiator bearing azo and halogen-containing ATRP initiating groups was first used to initiate the conventional free radical homopolymerization of phenoxyallene with a cumulated double bond to give a PPOA-based macroinitiator with ATRP-initiating groups at both ends. PMMA-*b*-PPOA-*b*-PMMA and PBA-*b*-PPOA-*b*-PBA triblock copolymers were synthesized by the ATRP of methyl methacrylate and *n*-butyl acrylate, as depicted in Scheme 2.

RAFT, on the other hand, has seen rapid growth due to its superior compatibility with a broader range of functionalities and high tolerance of impurities. The creative combination of RAFT polymerization with other polymerization techniques, such as ATRP or ring-opening polymerization, has extended the array of available architectures such as graft, star, hyperbranched, etc. [31,32]. The general method for the preparation of BCPs using the RAFT process is through sequential polymerization. For the synthesis of an AB diblock copolymer, the first block was synthesized via a RAFT process, followed by subsequent purification. The resulting end-reactive polymer acts as a macro-RAFT agent for a second polymerization step. To ensure complete end group functionalization, the polymerization yield of the first block is usually kept rather low (<30%). In contrast to this two-step process, Chaduc et al. reported a simple one-pot RAFT process for amphiphilic block copolymers in water [33]. Using 4-cyano-4-thiothiopropylsulfanyl pentanoic acid (CTPPA) as the RAFT agent, poly(acrylic acid), poly(methacrylic acid), or poly(methacrylic acid-*co*-poly(ethylene oxide) methyl ether methacrylate) was first formed in water. The resulting macro-RAFT agents were then directly used without further purification for the RAFT polymerization of styrene in water in the same reactor. This strategy leads to a very good control of the resulting amphiphilic block copolymers. Very recently, short poly(ethylene glycol) (PEG) was employed as the solvent in the macromolecular RAFT agent-mediated dispersion polymerization of BCPs such as PEG-*b*-PS, P4VP-*b*-PS, and PNIPAM-*b*-PS. A new formulation of polymerization-induced self-assembly of PEG named PEG-PISA to synthesize diblock copolymer nanoassemblies was reported. In PEG-PISA, the viscous PEG affords advantages including fast polymerization rate, good control over the synthesis of diblock copolymers, and in situ synthesis of both amphiphilic and doubly hydrophobic diblock copolymer nanoassemblies at a polymer concentration of up to 50%. Furthermore, two new self-assembled morphologies of ellipsoidal vesicles and nanotubes were formed via PEG-PISA. Nevertheless, RAFT dispersion polymerization often suffers from rather poor colloidal stability, leading to low polymerization rate, broad molecular weight distribution, and lack of control over molecular weight when using a small RAFT agent. The reason for this is believed to be the superswelling effect in the early nucleation stage [34]. Zhu et al. employed an amphiphilic oligomer, poly(acrylic acid-*b*-styrene) trithiocarbonate, as both surfactant and RAFT agent to polymerize styrene. This macro-RAFT agent was capable of enhancing the colloidal stability and, as a result, the polymerization was successful [34,35]. Well-controlled PS with a molecular weight up to 120 kg/mol and PS-*b*-PBA were readily synthesized using this method. Furthermore, the same group demonstrated that this method could be used as a promising approach to synthesize thermoplastic elastomer materials [36].

**Scheme 3:**
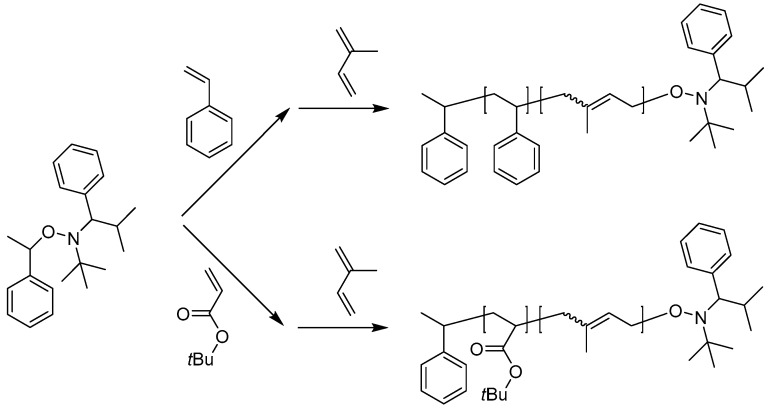
Synthetic scheme of PI-*b*-P*t*BuA and PS-*b*-PI block copolymers using a modified nitroxide-mediated polymerization (NMP) initiator.

NMP is commonly used for the synthesis of block copolymers consisting of PS, PMMA, poly(vinyl acetate) (PVAc) and poly(dimethyl acrylamide) (PDMA). In a typical process, styrene is polymerized firstly using a bi-molecular initiator benzoyl peroxide and 2,2,6,6-tetramethylpiperidinoxy (TEMPO). The PS segment bearing the TEMPO end group is subsequently used as the macroinitiator for the second block. While the molecular weight distributions are usually broad, this may not limit their practical applications. The monomer conversion is also low when polymerized at high temperature. The deviation from the target molecular weight became more significant, especially when it became higher than 100,000 g/mol. Hawker et al. reported a modified NMP system for the controlled polymerization of dienes in the presence of alkoxyamine initiators based on a 2,2,5-trimethyl-4-phenyl-3-azahexane-3-oxy skeleton (Scheme 3). A detailed study revealed that this modified system was able to control the homopolymerization of isoprene to high conversion and molecular weights from 1000 to 100 000 g/mol with polydispersities of 1.06–1.15. Polyisoprene-containing BCPs, PI-*b*-P*t*BuA and PS-*b*-PI, were also prepared with similar control. In comparison with conventional TEMPO systems, these new systems exhibit a significant improvement in the ability to control the polymerization and further demonstrate the versatility of nitroxide-mediated living free radical procedures [37]. 

#### 2.1.2. Living Anionic Polymerization

The pioneering work on BCP synthesis using living anionic polymerization (LAP) was reported by Szwarc et al. in 1956 [38,39]. This type of polymerization proceeds to quantitative conversion without chain transfer and/or chain termination. Although there might be an argument that the demanding experimental conditions is one of its limitations, LAP has proved itself to be the best polymerization technique for the preparation of well-defined BCPs based on vinyl monomers such as styrene, dienes, (meth)acrylates, vinyl pyridines, acrylonitriles, as well as cyclic monomers such as lactones, oxiranes, and siloxanes over the last 60 years [2,40,41]. Anionic polymerization is widely exploited in industry to create BCPs on a massive scale. Because of its superior control over the molecular weight, architecture, composition, and functionality as compared to controlled radical polymerization, almost all well-defined BCPs with complex architectures such as star, comb, graft, dendritic, etc. are achievable via the combination of anionic polymerization and linking chemistry [6,42,43,44,45,46,47]. There are several insightful reviews available that describe the recent progress in the synthesis of well-defined BCPs via LAP [48,49]. 

**Scheme 4:**
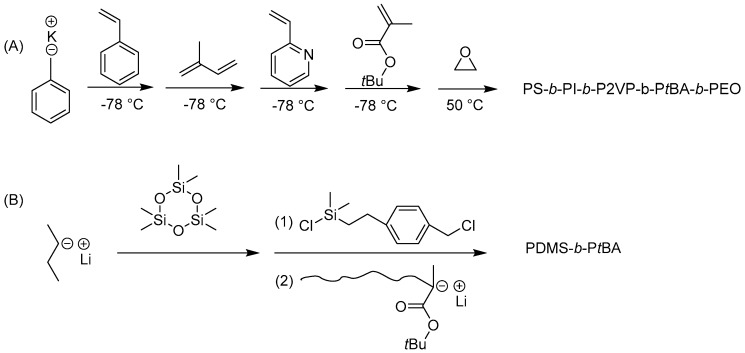
(**A**) Synthesis of PS-*b*-PI-*b*-P2VP-*b*-P*t*BA-*b*-PEO pentablock copolymers via sequential living anionic polymerization (LAP). (**B**) Synthesis of PDMS-*b*-P*t*BA diblock copolymers.

One key condition that must be satisfied is that the nucleophilicity of the macroanion must be sufficiently high to initiate the second monomer. Thus, the monomers must be added in the order of increasing electron affinity: styrene < butadiene ~ isoprene < vinyl pyridine < methyl acrylate < ethylene oxide. One classic example of the synthesis of multiblock copolymers by sequential polymerization was reported by Hadjichristidis et al., as shown in Scheme 4A [50]. The resulting BCPs exhibited a very low PDI of 1.04 and the compositions observed from ^1^H-NMR were consistent with the feeding ratios.

**Figure 3:**
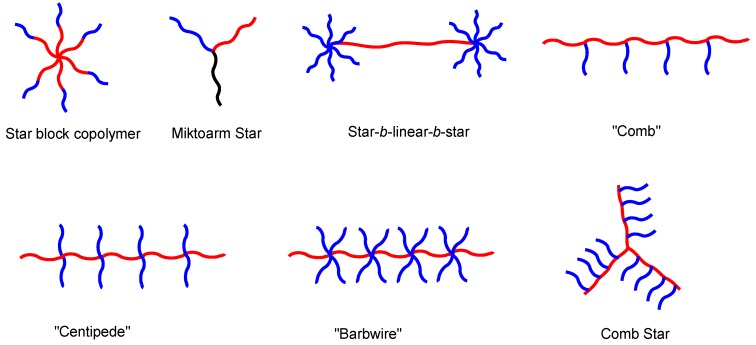
Illustration of complex architectures using living anionic polymerization and coupling chemistry. Adapted from Reference [55]. (Copyright (2017) WILEY-VCH Verlag GmbH & Co. KGaA, Weinheim).

Nevertheless, sequential LAP is restricted especially when the monomers exhibit different reactivities. For example, the living polymer of polydimethylsiloxane is not sufficiently nucleophilic to initiate the polymerization of *t*-butyl methacrylate. In order to prepare PDMS-*b*-P*t*BA, a linking reagent bearing with chlorosilane moiety was used due to its high reactivity (Scheme 4B) [51]. Hirao et al. prepared a wide variety of block copolymers with extremely low PDI using new linking methodology. Another advantage of this linking chemistry is that it opens endless possibilities of various molecular architectures [5,42,52,53], as demonstrated by the work of Mays, Hadjichristidis, Hirao, Quirk, and many others through chlorosilane and 1,1-diphenylethylene (DPE)-based linking chemistry (Figure 3). Well-defined BCPs with structural and compositional homogeneity prepared using LAP have served as templates for structure-property relationship studies and have also found many important industrial applications such as adhesives and sealants, in automotive, wire, paving, and footwear applications, etc. [54].

### 2.2. Combination of Different Polymerization Techniques

**Scheme 5:**
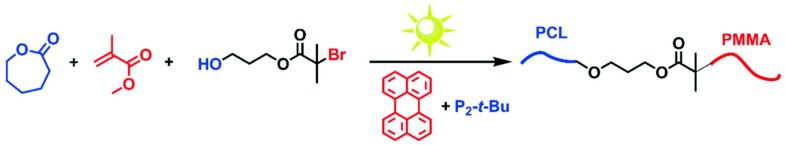
One pot polymerization of PMMA-*b*-PCL using combination of metal-free ATRP and ring opening polymerization (ROP) simultaneously under sunlight. Reprinted from Reference [56]. (Copyright (2017) Royal Society of Chemisty).

In some cases, sequential addition polymerization techniques are limited when the monomers are not suitable to be polymerized using the same polymerization mechanism. A second approach to BCPs is the coupling of two different polymerization techniques or block copolymer segments either using a “click” reaction such as Diels–Alder cycloaddition reaction, a thiol-ene reaction, or a copper-catalyzed azide–alkyne cycloaddition reaction (CuAAC). With appropriate care, this approach can yield BCPs without substantial homopolymers and comparable PDIs as compared with the sequential addition approach. Yagci et al. reported a one-pot synthetic approach to BCPs using the combination of ATRP and ring opening polymerization (ROP) methods simultaneously [56]. Two structurally different monomers were selected and the polymerization was initiated simultaneously using a difunctional initiator, as shown in Scheme 5. They found that the two polymerization mechanisms proceeded without affecting each other. The obtained products showed characteristic thermal transitions of both PMMA and polycaprolactone (PCL) blocks, indicating that BCPs were synthesized.

**Figure 4:**
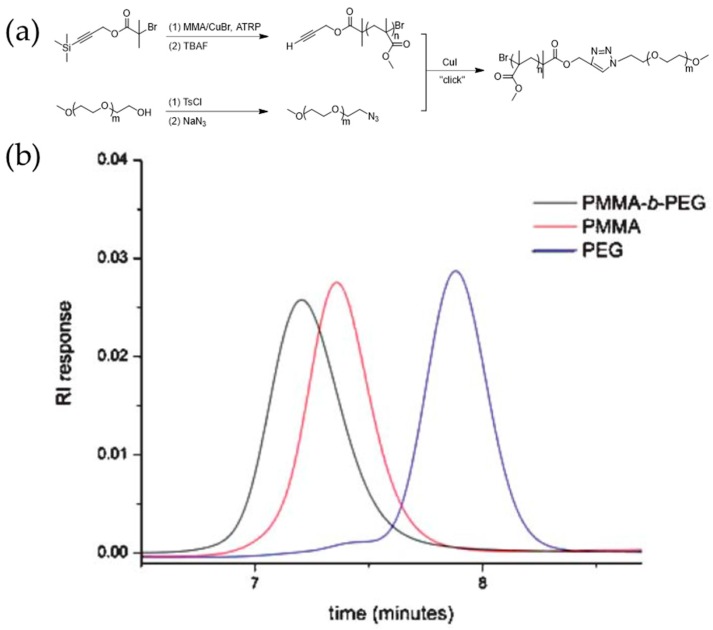
(**a**) Preparation of PMMA-*b*-PEG block copolymer via ATRP and azide-alkyne click reaction. (**b**) Size exclusion chromatography (SEC) curves of PMMA, polyethylene glycol (PEG), and PMMA-*b*-PEG diblock copolymer. Reprinted from Reference [57]. (Copyright (2005) Royal Society of Chemistry).

The synthesis of complex polymeric materials through post-polymerization coupling reactions has also attracted significant research interest. Van Hest et al. synthesized PMMA-*b*-PEG diblock copolymers via the combination of ATRP and alkyne-azide click reaction, as shown in Figure 4. Terminal alkyne and azide moieties were conveniently introduced via protected functionalized initiators [57].

**Figure 5:**
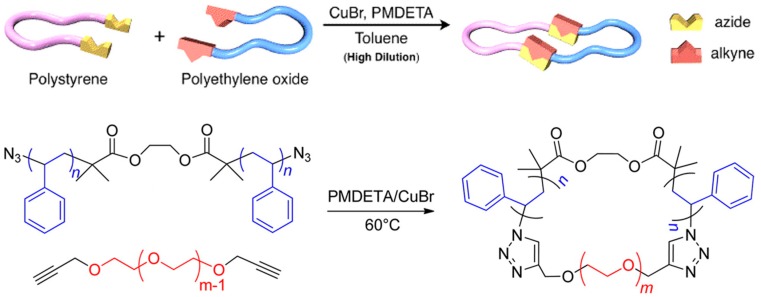
Synthesis of cyclic PS-*b*-PEO copolymer. Reprinted from Reference [11]. (Copyright (2012) American Chemical Society).

Hawker et al. synthesized a series of cyclic PS-*b*-PEO BCPs using the ATRP and CuAAC of α,ω-azide-functionalized PS and α,ω-alkyne PEO homopolymers (Figure 5) [11]. The α,ω-azide-functionalized PS was polymerized using a difunctional ATRP initiator, ethylene bis-(2-bromoisobutyrate). The α,ω-Br-terminated PS was then conveniently converted to the azide moiety. The α,ω-alkyne PEO homopolymer was obtained by treating α,ω-hydroxyl PEO with propargyl bromide. The desired products were isolated using preparative gel permeation chromatography. Tezuka et al. reviewed interesting work about topological studies of BCPs [58]. 

**Figure 6:**
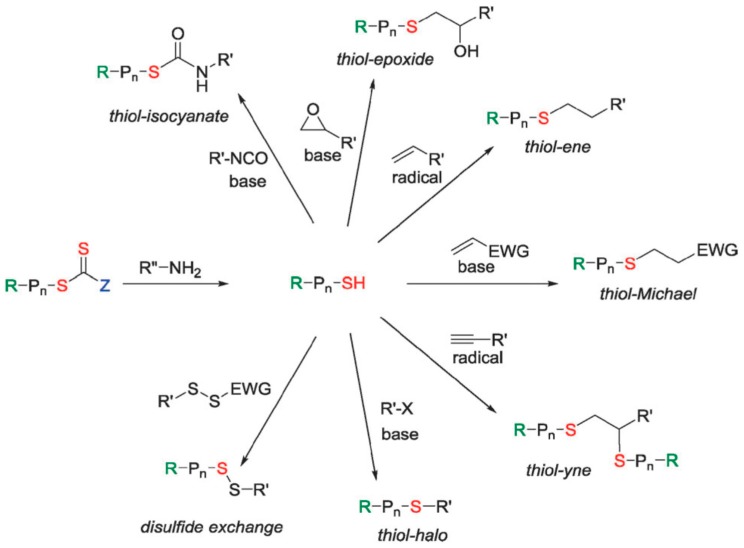
Synthetic strategies to block copolymers (BCPs) using thiol-based coupling reactions. Reprinted from Reference [59]. (Copyright (2014) Royal Society of Chemistry).

RAFT polymers can be conveniently converted to thiols by treating the chain end with aliphatic amines. A block copolymer can therefore be obtained through related thiol chemistry (Figure 6).

**Scheme 6:**
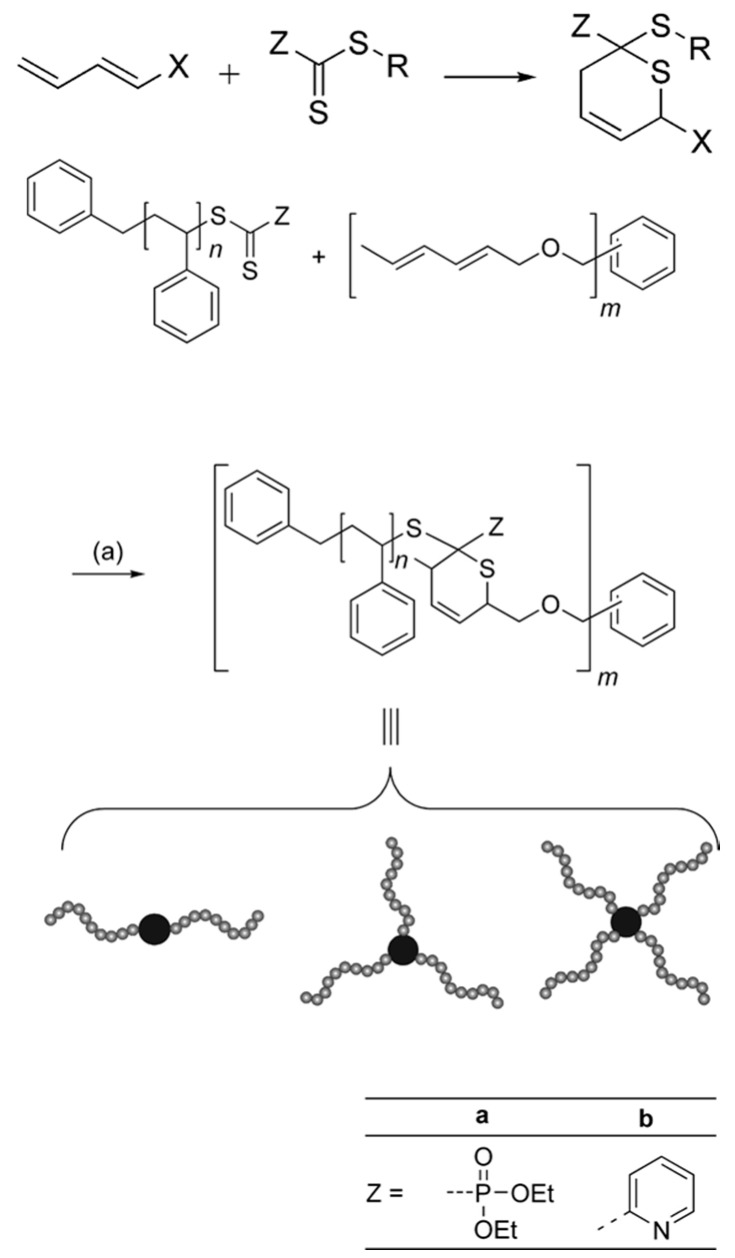
Formation of block copolymers via a reversible addition-fragmentation chain transfer (RAFT) polymerization and a hetero Diels-Alder reaction. Adapted from Reference [60]. (Copyright (2008) American Chemical Society).

Barner-Kowollik and Stenzel et al. first synthesized a series of star polymers by a combination of RAFT chemistry and hetero Diels-Alder reaction [60], in which the reaction occurs between thiocarbonyl group and a diene in a [4 + 2] cycloaddition process (Scheme 6). 

The incorporation of supramolecular chemistry also provides a very interesting pathway to form BCPs. Schubert et al. demonstrated that terpyridine-terminated PS and PEO can form BCPs with RuCl_3_ [61]. The coordination junction point was formed in two steps: the terpyridine-terminated PEO was selectively complexed with RuCl_3_ yielding monocomplex of Ru(III); terpyridine-terminated PS was then reacted with the monocomplex under the reduction of Ru(III) to Ru(II), resulting in PS-*b*-PEO. One advantage of this approach is that the non-covalent junction point can be broken under external stimuli. 

## 3. Self-Assembly of BCPs

### 3.1. Self-Assembly in Bulk

**Figure 7:**
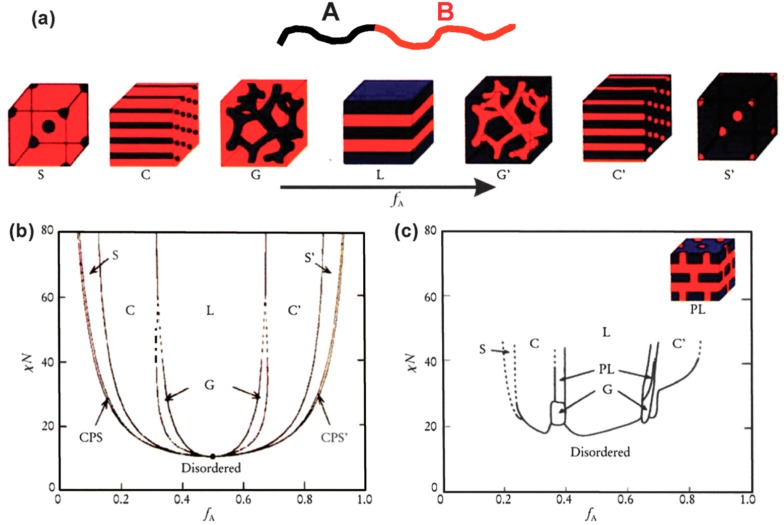
(**a**) Equilibrium morphologies of AB diblock copolymers in bulk: S and S’ = body-centered-cubic spheres, C and C’ = hexagonally packed cylinders, G and G’ = bicontinuous gyroids, and L = lamellae. (**b**) Theoretical phase diagram of AB diblocks predicted by the self-consistent mean-field theory, depending on volume fraction (*f*) of the blocks and the segregation parameter, χN; CPS and CPS’ = closely packed spheres. (**c**) Experimental phase diagram of polystyrene-*b*-polyisoprene copolymers, in which *f*_A_ represents the volume fraction of polyisoprene, PL = perforated lamellae. Reproduced from Reference [69]. (Copyright (2012) Royal Society of Chemistry).

Self-assembly of BCPs with immiscible blocks has been extensively studied via experiments and simulations since the 1960s [62]. Several reviews are available on this topic [22,63,64]. The self-assembly process is driven by an unfavorable mixing enthalpy coupled with a small mixing entropy. Composition (*f*), the number of repeating units (N), and the Flory-Huggins interaction parameter (χ) are the important parameters that determine the morphologies, which include spheres (S), cylinders (C), gyroids (G), and lamellae (L) [65,66]. The self-assembly behavior may also be influenced by other external parameters such as mechanical or electric fields [67,68]. However, it is beyond the scope of this review to cover all these aspects. Therefore, we only address the basic concepts of microphase separation and present several recent examples of the morphological behavior of BCPs. Figure 7 shows that the different morphologies of a typical linear diblock BCP evolve from spherical to lamellar and can undergo disorder to order transitions, as a function of *f* and χN. 

**Figure 8:**
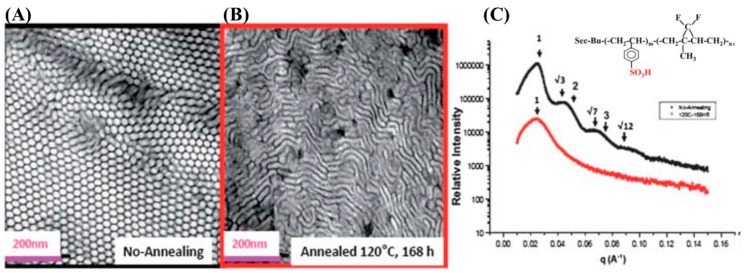
TEM and small-angle X-ray scattering (SAXS) images of *s*PS-*b*-*f*PI. (**A**) ordered hexagonal structures without annealing; (**B**) less ordered structures of sample annealed at 120 °C; (**C**) SAXS of the corresponding samples in (**A**,**B**). Reprinted from Reference [72]. (Copyright (2010) Royal Society of Chemistry).

Furthermore, the self-assembly of triblock copolymers of poly(styrene-*b*-isoprene-*b*-styrene) (PS-*b*-PI-*b*-PS) has also been extensively investigated using small-angle X-ray scattering (SAXS) and transmission electron microscopy (TEM) by Storey et al. and Elabd et al. [70,71]. Unlike the conventional study on neutral BCPs, Mays et al. compared the morphologies of well-defined neutral PS-*b*-PI and its charged counterpart, sulfonated PS-*b*-fluorinated PI (*s*PS-*b*-*f*PI). Interestingly, Inversed morphologies were observed when the diblock BCP, 25*s*PS-*b*-75*f*PI (50% of PS block was sulfonated to sulfonic form), was casted from tetrahydrofuran compared with its neutral counterpart, as shown in Figure 8 [72]. 

Segalman et al. prepared a series of ionic conducting BCPs to investigate the relationship between ionic conductivity and domain spacing [73]. A well-defined BCP precursor, PS-*b*-PI, was synthesized followed by a thiol-ene reaction to attach the ionic moieties to PI segments. The domain spacing was controlled by keeping the volume fraction of ionic moieties constant and varying the BCP chain length. It was revealed that the ionic conductivity was independent of domain spacing. The insights gained by this work could facilitate the development of design rules for the next generation of high performance ion-conducting membranes. Balsara et al. studied the effect of salt on the morphology of electron conducting poly(3-(2′-ethylhexyl)thiophene)-*b*-ethylene oxide) (P3EHT-*b*-PEO) [74]. PEO was selected due to its good salt solvation capability. Their results showed that, in the melt state, the salt-free sample exhibits a gyroid morphology while the salt containing sample (r = 0.125) exhibits a lamellar morphology. Furthermore, quenching the salt-free sample to room temperature results in a lamellar morphology due to the breakout of the P3EHT crystals. In contrast, there is little change in the morphology of the salt-containing sample upon quenching to room temperature. This could be attributed to the increase in χN.

**Figure 9:**
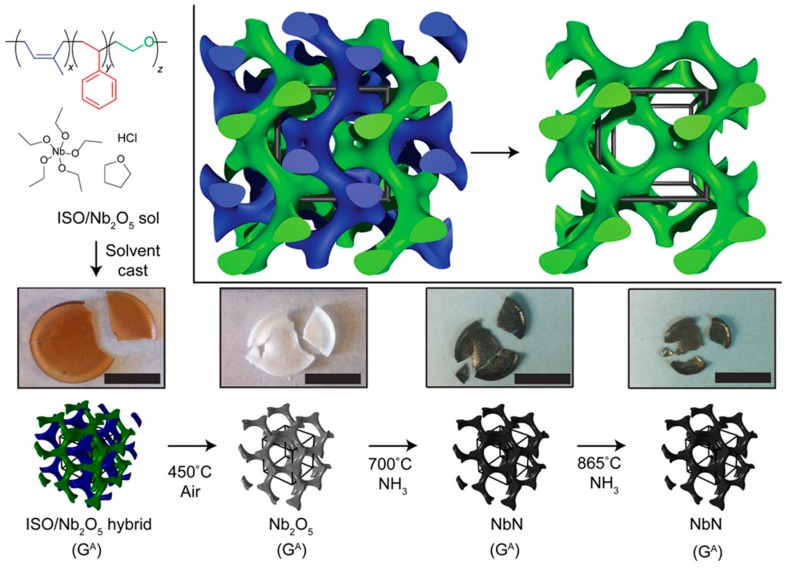
Gyroid-forming PI-*b*-PS-*b*-PEO block copolymers and the preparation process of NbN superconductors. Reprinted with permission from Reference [75]. (Copyright (2016) the authors).

Owing to its structural versatility and precise tunability of morphology, dimensionality, and feature size, BCP is an ideal platform for studying periodically ordered functional materials on the mesoscale. Wiesner et al. demonstrated the utilization of triblock copolymer (PI-*b*-PS-*b*-PEO) self-assembly to direct the synthesis of a mesoporous niobium nitride (NbN) superconductor (Figure 9). The formation of a three-dimensionally continuous gyroidal mesoporous NbN superconductor exhibits a critical temperature (T_c_) of about 7 to 8 K, a flux exclusion of about 5% compared to a dense NbN solid [75]. More examples will be given in Section 4.

The myriad morphologies that are formed by BCPs are usually obtained by slow cooling so that the polymer chains have enough time to reach thermodynamically preferred alignments. Rather than using slow cooling, Kim et al. rapidly quenched poly(isoprene-*b*-lactide) diblock copolymers from the disordered state and revealed an extraordinary thermal history dependence [76]. Whereas conventional cooling results in the formation of documented morphologies, rapidly cooled samples that are then heated from low temperature form the hexagonal C14 and cubic C15 Laves phases commonly found in metal alloys. This unusual discovery reinforces fundamental analogies between the way metals and self-assembled soft materials break symmetry when subjected to changes in thermodynamic state variables that drive phase transitions.

### 3.2. Self-Assembly in Solution

**Figure 10:**
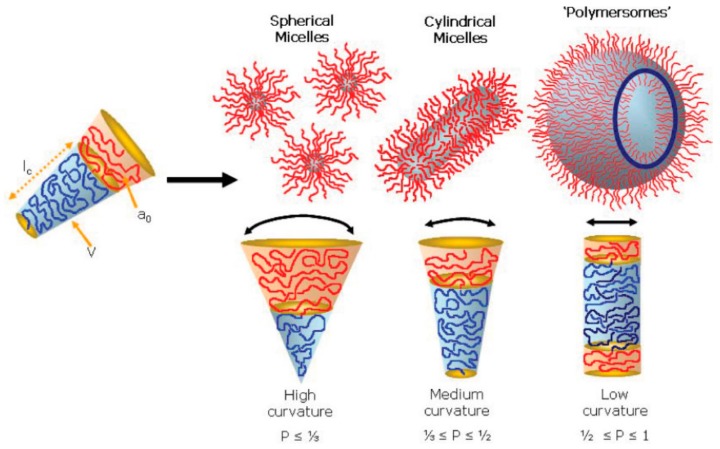
The types of formed nanostructures of amphiphilic diblock copolymers due to the inherent curvature of the polymer, as estimated by chain packing parameter, p. Reprinted from Reference [80]. (Copyright (2009) WILEY-VCH Verlag GmbH & Co. KGaA, Weinheim).

The self-assembly of amphiphilic BCPs has been a popular topic in fields such as nano-cargo delivery, biomedical/pharmaceutics, and nanotechnology [77,78,79]. Although the self-assembly of amphiphilic BCPs is also driven by the minimization of free energy in the system, self-assembly in solution is more complicated than BCP self-assembly in bulk. The morphologies are primarily determined by the so-called packing parameter (p = v/a_0_l_c_), where v is the volume of the hydrophobic chain, a_0_ is the optimal area of the head group, and l_c_ is the length of the hydrophobic tail (Figure 10) [80]. 

**Figure 11:**
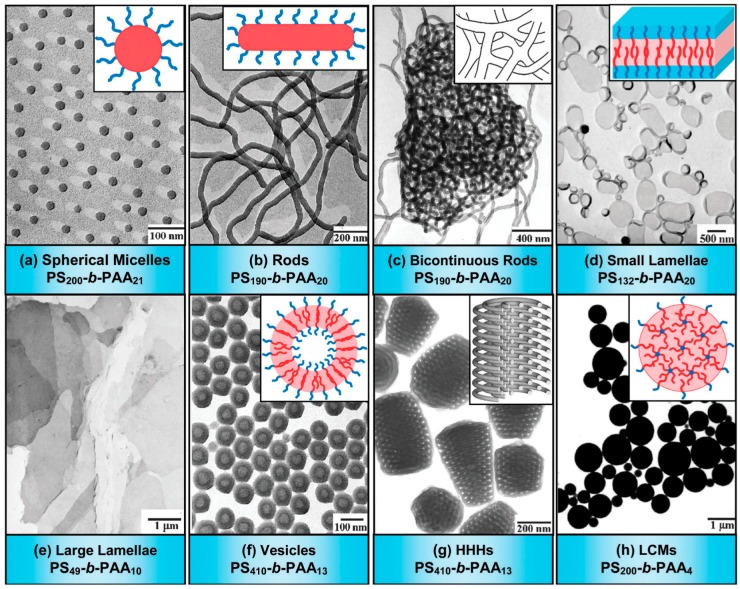
TEM images and corresponding schematic diagrams of various morphologies formed by amphiphilic PS_m_-*b*-PAA_n_ copolymers( m and n denote the degrees of polymerization of PS and PAA, respectively): (**a**) spherical micelles; (**b**) rods; (**c**) bicontinuous rods; (**d**) small lamellae; (**e**) large lamellae; (**f**) vesicles; (**g**) hexagonally packed hollow hoops (HHHs); (**h**) large compound micelles (LCMs). Reprinted from Reference [69]. (Copyright (2012) Royal Society of Chemistry).

By controlling the factors that are able to change the packing parameter such as BCP composition and concentration, water content, common solvent, and additives, a wide range of morphologies have been reported including spherical micelles, cylindrical, bi-continuous, lamella, vesicles, tubules, etc. [81] (Figure 11).

**Figure 12:**
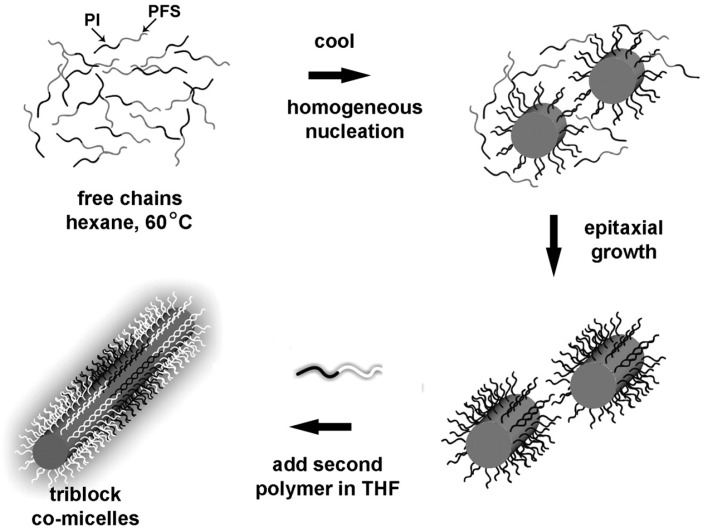
The schematic representation of the micelles from polyferrocenylsilane (PFS)—containing BCPs via epitaxial growth. Reprinted from Reference [82]. (Copyright (2007) American Association for the Advancement of Science).

Manners and Winnik reported a series of interesting rod-like micelles using poly(ferrocenyldimethylsilane) (PFDMS). These micelles are able to grow epitaxially by the addition of more polymer, leading to extended micelles with a very narrow size distribution (Figure 12) [82].

**Figure 13:**
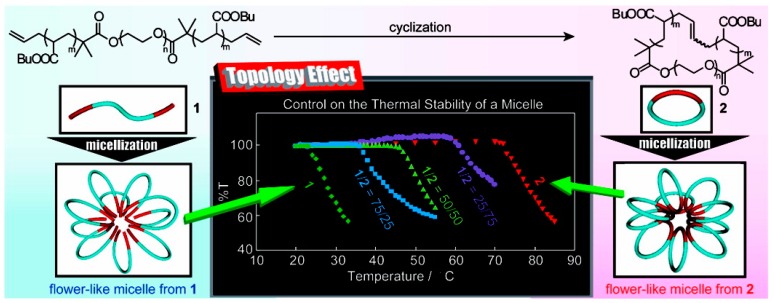
The schematic illustration of linear amphiphilic PBA-*b*-PEO-*b*-PBA and cyclic PBA-*b*-PEO-*b*-PBA self-assembly in aqueous media. The cyclic BCP shows an increased T_c_. Reprinted from Reference [83]. (Copyright (2010) American Chemical Society).

Self-assembly of BCPs enables researchers to construct nanoscale structures with molecular level precision. Not only the chemical composition has significant effect on the properties of nanostructure; molecular architecture also has profound effect, as revealed by the recent studies. Tezuka and Yamamoto et al. explored the effect of topology on the thermal stability of self-assembled structures of BCPs: linear poly(butyl acrylate-*b*-ethylene oxide-*b*-butyl acrylate) (PBA-*b*-PEO-*b*-PBA) (1) and the cyclic counterpart (2) (Figure 13). Despite no distinctive change in the chemical composition or structure of the micelles, they found that the cloud point (T_c_) was increased by more than 40 °C through the topological conversion. Moreover, the T_c_ can be tuned by the changing the ratio of 1 and 2 [83].

**Figure 14:**
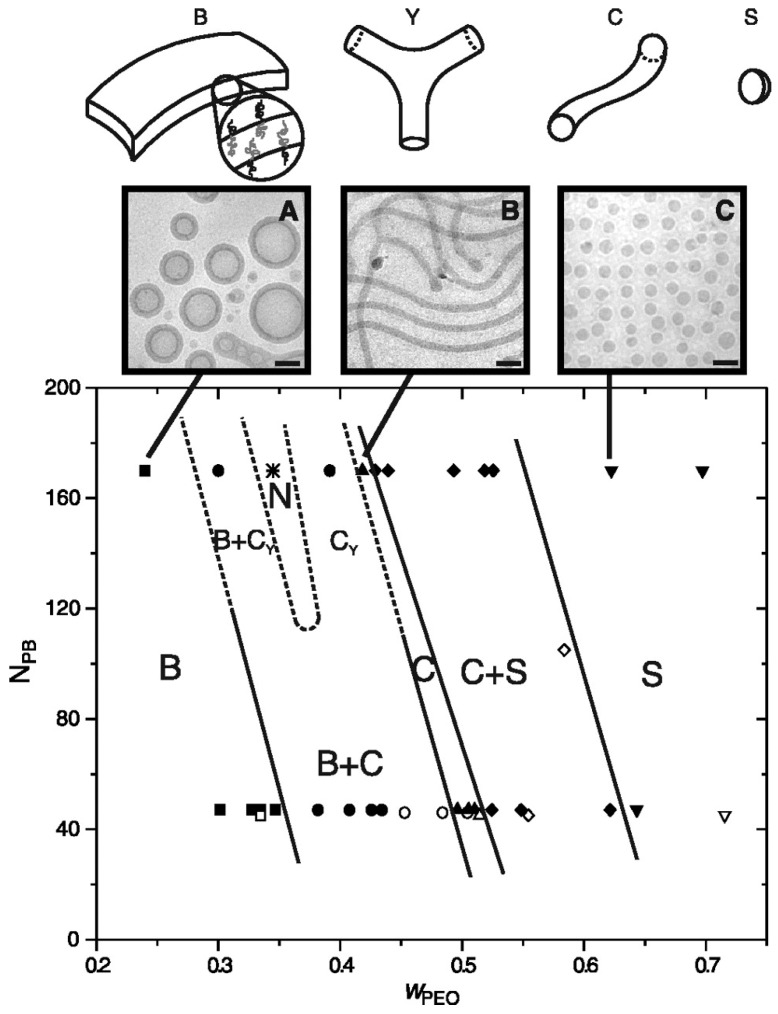
Morphology diagram for PB-PEO in water (1 wt %) as a function of molecular size and composition, where N_PB_ and W_PEO_ are the degree of polymerization and weight fraction of the PB and PEO blocks, respectively. Reprinted from Reference [84]. (Copyright (2003) American Association for the Advancement of Science).

Using poly(1,2-butadiene-*b*-ehtylene oxide) (PB-*b*-PEO) in water media, Bates et al. observed the formation of “Y-junctions”, which further assembled to form a 3D network (Figure 14). This observation is in contrast to that of the low molecular weight counterpart, where only spherical micelles, wormlike micelles, and vesicles were obtained when mixed with water [84]. 

## 4. Applications

### 4.1. Applications as Thermoplastic Elastomers

One of the most important technical applications of BCPs is as thermoplastic elastomers (TPEs). This type of BCP typically contains physically crosslinked rigid glassy domains and a continuous soft rubbery domain. It offers the elasticity of conventional rubber and, since it is not chemically crosslinked, is suitable for typical plastic processes such as injection molding and melt extrusion. TPEs have found applications in adhesives, coatings, food packaging, and many other areas [85]. The most commonly studied TPEs are poly(styrene-*b*-isoprene-*b*-styrene) (SIS) and poly(styrene-*b*-butadiene-*b*-styrene) (SBS). These two types of BCPs were firstly developed by Holden and Milkovich through living anionic polymerization in the early 1960s [86]. The remarkable mechanical properties are attributed to the microphase separation of the two chemically incompatible blocks, PS and PI (or PB). SIS with high 3,4-isoprene exhibits a broad glass transition close to room temperature, providing a very good vibration damping material at room temperature [54]. Polyethylene (PE) or poly(ethylene-alt-propylene) can be obtained through the hydrogenation of the diene block; such products exhibit superior stability under light and oxygen. Hydrogenated SBS has been used in applications such as electrical wire and cable sheathing due to its cold temperature flexibility and higher extendibility to incorporate flame retardants over conventional poly(vinyl chloride) (PVC) [54]. Star-block copolymers of butadiene and styrene have found applications in adhesives and sealants [87]. 

**Figure 15:**
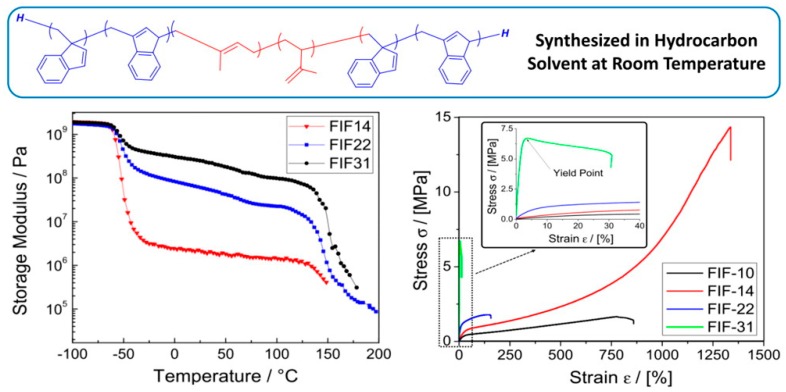
The structure of poly(benzofulvene (BF)-*b*-isoprene-*b*-benzofulvene) (FIF) block copolymer and mechanical analysis. Reprinted from Reference [92]. (Copyright (2016) American Chemical Society).

Because the precise control over composition and architecture is possible through LAP, the resulting BCPs can be tailored to a wide variety of applications [88]. One research focus is to improve the upper service temperature of styrenic thermoplastic elastomers (S-TPEs), which is mainly controlled by the T_g_ of the hard domain. Considerable research has focused on developing high T_g_ glassy domains such as poly(α-methyl styrene) (T_g_ ~ 173 °C) [89], poly(α-methyl *p*-methyl styrene) (T_g_ ~ 183 °C) [90], and poly(*tert*-butyl styrene) (P*t*BS, T_g_ ~ 130 °C) [91]. In hydrocarbon solvent at room temperature, Mays et al. prepared a series of BCPs consisting of poly(benzofulvene (BF)-*b*-isoprene-*b*-benzofulvene) (FIF), in which FIF with 14 vol % of PBF exhibited a maximum stress of 14.3 ± 1.3 MPa and strain at break of 1390 ± 66% from tensile tests [92]. Dynamic mechanical analysis showed that the softening temperature of PBF in FIF was 145 °C, which is much higher than that of thermoplastic elastomers with PS hard blocks (Figure 15). 

In comparison with diene-based TPEs, all acrylic-based TPEs showed higher oxidation stability and UV resistance. The same group synthesized a series of TP all acrylic-based high temperature thermoplastic elastomers containing poly(1-adamatyl acrylate) as a hard domain and poly(tetrahydrofurfuryl acrylate) as a soft domain by RAFT polymerization [93]. These TPEs exhibited superior stress-strain behavior compared to that of conventional all acrylic-based TPEs consisting of PMMA and PBA made by controlled radical processes [94], while the tensile strength was lower compared to that of similar products prepared via living anionic polymerization [95].

**Figure 16:**
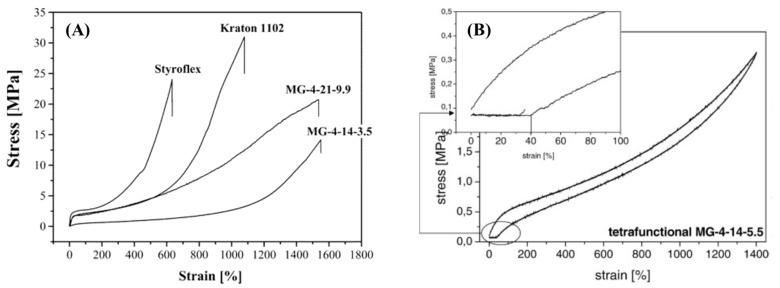
(**A**) Stress–strain behavior of tetrafunctional multigraft copolymers compared to commercial thermoplastic elastomers. Reprinted from Reference [96]. (Copyright (2006) American Chemical Society). (**B**) Hysteresis curve of a tetrafunctional multigraft copolymer with 14 vol % PS and 5.5 branching points. Reprinted from Reference [97]. (Copyright (2006) WILEY-VCH Verlag GmbH & Co. KGaA, Weinheim).

TPEs with non-linear architectures have also been synthesized and investigated over the last three decades. Multigraft copolymers of PI-*g*-PS are found to have high tensile strength, high strain at break, and low residue strain. Furthermore, Mays et al. synthesized a series of tetrafunctional multigraft copolymer (“centipede”) with varied branching points (Figure 16) [96]. The tetrafunctional copolymer with 10 branching points showed an exceptional elongation at break ~2100%, almost double that of commercial Kraton^®^ (1050% at similar composition), and only 40% residual strain on hysteresis experiments (elongated at 1400%) [97]. Detailed study revealed that the superelasticity was attributed to the distinct molecular architectures. Furthermore, the same group developed a low-cost emulsion polymerization to prepare comb multigraft copolymers such as PI-*g*-PS and PBA-*g*-PS as well as centipede PBA-*g*-PS for TPE applications [98,99]. 

### 4.2. Applications in Drug Delivery and Release

Advanced nanoscale systems created by self-assembly of BCPs have seen tremendous progress for drug delivery applications over the last decade [100,101]. The advances in BCP self-assembly offer effective control of morphology, surface chemistry, and the introduction of environmental responsiveness. Among these nanostructures, micelles and vesicles are the most studied morphologies. Block copolymer micelles are of interest for drug delivery applications for a number of reasons. First of all, hydrophobic drugs can be entrapped in the core and transported at concentrations that can exceed their intrinsic water solubility. Secondly, the hydrophilic blocks, which are often composed of PEO, can form hydrogen bonds with the aqueous surroundings and form a tight shell around the micellar core. Moreover, the PEO corona resists protein adsorption and cellular adhesion, protecting the hydrophobic drug against hydrolysis and enzymatic degradation. In addition, the PEO corona prevents recognition by the reticuloendothelial system and therefore increases the blood circulation times [100]. Thermo-responsive micelles are the most widely investigated, such as poly(*N*-isopropyl acrylamide-*b*-d,l-lactide), poly(*N*-isopropyl acrylamide-*b*-butyl methacrylate), and poly(*N*-isopropyl acrylamide-*co*-*N*,*N*-dimethylacrylamide)-*b*-poly(d,l-lactide-*co*-glycolide) [102,103]. These micelles undergo reversible structural changes that facilitate drug release once the temperature is elevated above the LCST of the polymers [104]. Other stimuli-responsive BCPs have also been extensively studied. Park et al. developed an amphiphilic BCP composed of PEG and poly(ε-(4-nitro)benzyloxycarbonyl-l-lysine) for hypoxia-sensitive drug delivery. The formed micelles encapsulated doxorubicin (DOX) in an aqueous condition and exhibited rapid intracellular release of DOX under the hypoxic condition [105]. 

BCPs used in drug delivery should have low toxicity or be non-toxic. BCP vesicles containing biocompatible PEO, PLA, and PCL have been evaluated [106,107]. Both PEO-*b*-PLA and PEO-*b*-PCL vesicles exhibit the controlled release of active dyes and anti-cancer drugs over periods of up to two weeks [108,109]. Discher et al. employed vesicles made from a mixture of biocompatible PB-*b*-PEO and PLA-*b*-PEO to simultaneously deliver two anti-cancer drugs, in which hydrophilic doxorubicin was encapsulated in the cavity and hydrophobic paclitaxel was embedded in the vesicle wall [106,110]. In vivo studies revealed that the vesicle-loaded drugs are more effective and sustainable in tumor shrinkage as compared with the direct injection of free drugs. Rates of encapsulant release from the hydrolyzable vesicles are accelerated with an increased proportion of PEG and the molar ratio of degradable copolymer. In particular, BCP vesicles modified with stimuli-responsive functionalities have been studied for smart drug delivery. Attaching the vesicle with antibodies allows the delivery of vesicles to targeted cells [111]. Other than their academic interest, thermosensitive liposomes (TSLs) have already been investigated for the treatment of breast cancer and colorectal liver metastasis in several clinical trials. 

In spite of the advances in the design of peptide drugs and the emergence of gene therapy, there is still a need to further improve the performance of these systems that can precisely direct the drug to the desired site in the body and to accurately control the rate at which the drug is released. 

### 4.3. Applications in Soft Lithography

**Figure 17:**
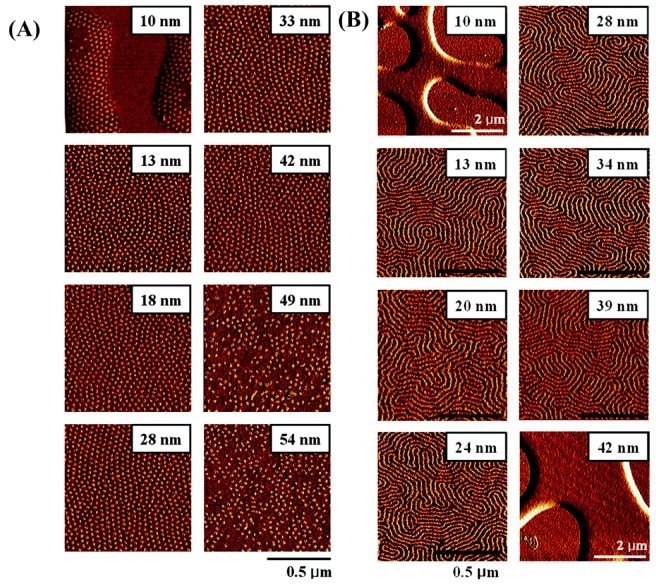
Surface force microscopy (SFM) phase image of (**A**) cylinder-forming PS-*b*-PMMA and (**B**) lamella-forming PS-*b*-PMMA on the substrate modified by R64 (PS mole fraction of 0.64) and R55 (PS mole fraction of 0.55), respectively, at various film thicknesses of block copolymer after thermally annealing thin films at 170 °C for 24 h. Reprinted from Reference [119]. (Copyright (2008) American Chemical Society).

One of the remarkable candidates for soft lithography is the directed self-assembly (DSA) of BCPs, because they can form ordered features at a length scale as low as a few nanometers, which is required for many of the most demanding next generation patterning applications, including the fabrication of bit patterned media (BPM) for hard disk drives as well as fin field effect transistor (FinFETs) and contact holes for microelectronics [112,113,114,115,116,117,118]. Poly(styrene-*b*-methyl methacrylate) (PS-*b*-PMMA) is the most studied BCP system because it can produce lamellar or cylinder morphologies, which are perpendicularly oriented to the substrate simply via thermal treatment (Figure 17) [119]. This phenomenon is attributed to the similarity of the interfacial energies between each block and the air interface [120]. Hawker et al. reported that the neutral surface (γPS-Air ≈ γPMMA-Air) could be achieved with thermally annealed PS-*b*-PMMA at 225 °C in air [121].

**Figure 18:**
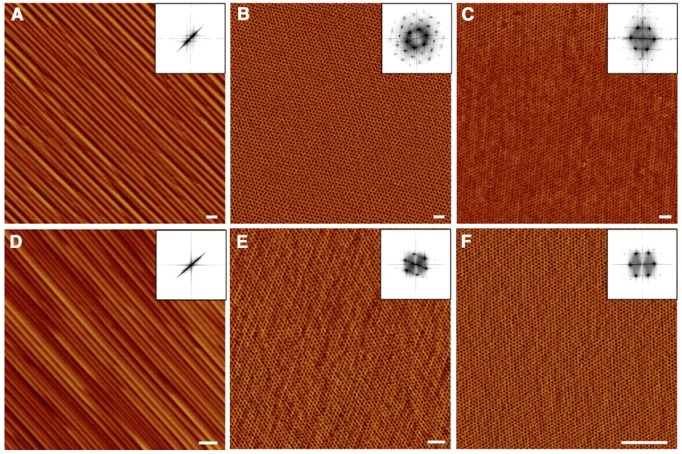
Atomic force microscopy (AFM) height images of sawtooth patterns and phase images of solvent-annealed PS-*b*-PEO thin films. (**A**,**D**) When the M-plane sapphire was annealed at 1400 °C and 1500 °C, a pitch of ~48 and ~24 nm and a peak-to-valley depth of ~6 and ~3 nm were obtained, respectively. Highly ordered PEO cylindrical microdomains having areal densities of 0.74 to 10.5 terabit/inch2 from PS-*b*-PEO (*M*_n_ = 26.5 kg/mol) (**B**), PS-*b*-PEO (*M*_n_ = 25.4 kg/mol) (**C**), PS-*b*-PEO (*M*_n_ = 21.0 kg/mol) (**E**), and PS-*b*-PEO (*M*_n_ = 7.0 kg/mol) (**F**) BCP thin films annealed in o-xylene vapor were obtained. Scale bars, 100 nm. Reprinted from Reference [123]. (Copyright (2009) American Association for the Advancement of Science).

Unfortunately, the minimum feature size is limited to ~13 nm due to its low χ (~0.039 at 150 °C) [122]. To achieve very high resolution (i.e., small feature size), the chemical structure of the BCP must be carefully designed to maximize the chemical incompatibility between blocks (i.e., high-χ, low-N). Impressive work has been reported employing a variety of high-χ BCPs. Russell et al. were able to achieve a 3-nm cylindrical domain size using PS-*b*-PEO on a surface of a sapphire single-crystal (Figure 18) [123]. 

Hillmyer et al. synthesized poly(lactide-*b*-dimethylsiloxane-lactide) (PLA-PDMS-PLA) to achieve a 7-nm pitch size [124]. Some other BCPs that are capable of self-assembling into sub-10 nm domain sizes include PS-*b*-PDMS [125], PS-*b*-P2VP [126], and PS-*b*-PLA [118]. On the other hand, high-χ organic BCPs that can form sub-10 nm domains suffer from low etch contrast when sizes approach down to 10 nm. Since organic polymers that contain inorganic constituents, such as silicon, are inherently etch resistant, the incorporation of one such block in high-χ BCPs affords exceptional etch contrast. Cushen et al. synthesized a series of high-χ BCPs composed of oligosaccharides coupled to a silicon-containing polystyrene derivative. The BCPs exhibit hexagonally packed cylinders with a 5-nm feature size in thin film [127]. A polarity-switchable top layer was also applied to the surface of silicon-containing BCPs films to obtain perpendicular orientation of poly(styrene-*b*-trimethyl silyl styrene-*b*-styrene) (PS-*b*-PTMSS-*b*-PS) and poly(trimethyl silyl styrene-*b*-lactide) (PTMSS-*b*-PLA) after thermal annealing [128]. 

**Figure 19:**
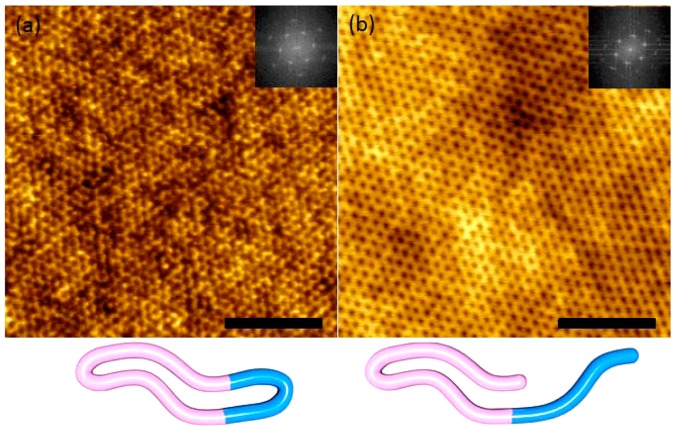
Atomic force microscopy height images for (**a**) cyclic PS13K-*b*-PEO5K and (**b**) PS13K-*b*-PEO5K diblock copolymers (inlet: 2D fast Fourier transform (FFT) of AFM images). Scale bars are 250 nm. Reprinted from Reference [11]. (Copyright (2012) American Chemical Society).

On the other hand, BCP architecture also plays a key role in controlling the feature size. Hawker compared linear PS-*b*-PEO and cyclic PS-*b*-PEO and found that the thin film self-assembly of the latter showed a ~30% decrease in domain spacing (Figure 19), which was attributed to the reduced hydrodynamic radii of the cyclic systems [11].

Limited synthetic access has largely restricted the application of cyclic BCPs in soft lithography. Other complex architecture such as star copolymers, etc. remains a largely unexplored regime. Nevertheless, the advancement of BCP design, synthesis, processing strategies, and morphological characterization tools (e.g., atomic force microscopy (AFM), tomographic TEM, grazing-incidence small-angle scattering (GISAXS), etc.) offers hope that this technology may impact industry and society in the coming years.

### 4.4. Applications in Mesoporous Materials

Research interest in ordered porous materials originated from the successful synthesis of porous silicates in the 1990s and soon spread to a variety of framework compositions including metal oxides, non-oxide inorganics, and carbon [129]. This group of materials exhibits periodically aligned structures and uniform cavities with sizes ranging from micro- (<2 nm), to meso- (2–50 nm), to macropores (>50 nm) [130], which lead to very high surface areas (up to 1500 m^2^/g) [131]. With these unique features, porous materials present great value for applications in energy conversion and storage [132,133,134,135], catalysis [136], drug delivery [137], gas capture [138,139], and water purification [140,141]. 

As mentioned in the previous section, the self-assembly of BCPs may induce a series of interesting 2D or 3D morphologies, which make them very useful as structure-directing “templates” for the fabrication of porous materials. Generally, the formation of porous materials through polymer templating involves the following steps: (1) Cooperative self-assembly of amphiphilic BCPs in the presence of a precursor for the framework (such as hydrolyzed silicate, metal oxides, or carbon source) where the precursor is directed to the hydrophilic domain through hydrogen bonding, ion pairing or hydrophilic interactions; (2) solidification/condensation/crosslinking/curing of the precursor to form the framework of the porous material; and (3) removal of the BCP template by solvent washing or thermal decomposition leaving spaces for pores. However, the specific procedures vary based on the final shape of the porous material, namely membranes, spheres, or monoliths, and detailed examples will be given below.

The most commonly used copolymer template are the commercial Pluronic surfactants, a group of poly(ethylene glycol-*b*-propylene glycol-*b*-ethylene glycol) (PEG-*b*-PPG-*b*-PEG) with limited options for lengths of polymer blocks. Alternatively, amphiphilic BCPs with wider choices for composition and molecular weight of the polymer segments are readily designed and synthesized in the laboratory through controlled/“living” polymerization techniques [142]. These synthesized BCPs usually contain poly(2-vinylpyridine) (P2VP), poly(4-vinylpyridine) (P4VP), or poly(ethylene oxide) (PEO) as the hydrophilic block, and PS, PMMA, PI, polyisobutylene (PIB), and polyacrylonitrile (PAN) as the hydrophobic block [143,144]. In addition to high versatility, BCPs synthesized in the laboratory show other merits as compared to commercial Pluronic copolymers. The contrast in hydrophilicity between the two selected blocks are usually higher than for the PEG and PPG pair in the Pluronics, which is beneficial for the self-assembly of polymer micelles in aqueous solution. Moreover, hydrophobic blocks such as PS and PMMA exhibit much higher glass transition temperatures than PPG, resulting in better stability of the self-assembled structures. In the case of copolymers containing PS and PAN, the hydrophobic segments can be converted to carbon residue in high yield in the carbonization step, which provides additional mechanical support to the mesoporous framework [145].

Evaporation-induced self-assembly (EISA) is widely used in the preparation of ordered mesoporous films and particles, which is especially important for oxides. In a typical EISA process, the cooperative self-assembly of a precursor with an amphiphilic template is induced by a concentration gradient of an organic solvent such as tetrahydrofuran (THF), chloroform, or dioxane. Different from a general solvent-annealing process, precursor molecules are crosslinked in EISA as the solvent evaporates, leaving no chance for structure refinement after it is formed [146,147]. Wiesner et al. synthesized mesostructured silicate/copolymer composite film through EISA with PS-*b*-PEO as a structure-directing agent in a mixture of THF and water [148]. A large variety of mesoporous metal oxides in the form of powder were synthesized through EISA using commercial Pluronics as a template [149]. In addition, mesoporous carbon films were prepared via EISA from a thermosetting precursor (phenol-formaldehyde system, resol, resorcinol, etc.) using a variety of BCP templates such as PS-*b*-P4VP, PEO-*b*-PS, PEO-*b*-PMMA, and PEO-*b*-PMMA-*b*-PS [143]. Recently, Wei and coworkers reported the synthesis of nitrogen-doped mesoporous carbon in the form of powder through EISA, using Pluoric copolymer as the template, with resol and dicyandiamide as sources for C and N, respectively [147]. For clarification, some research groups do not consider systems involving formaldehyde as an actual EISA process, since the crosslinking of precursors occurs after the cooperative self-assembly, instead of simultaneously, as in many EISA processes [146]. 

**Figure 20:**
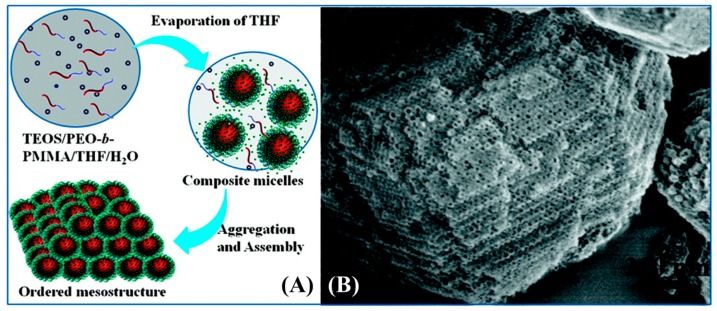
(**A**) Formation mechanism of ordered mesoporous silica through the solvent evaporation -induced aggregating assembly (EIAA) process by using diblock copolymer PEO-*b*-PMMA as a template, tetraethylorthosilicate (TEOS) as the silica source, and acidic tetrahydrofuran (THF)/H_2_O mixture as the solvent; (**B**) Typical field-emission scanning electron microscopy (FESEM) image of mesoporous silica calcinated in air at 550 °C. Reprinted from Reference [150]. (Copyright (2011) American Chemical Society).

Following the traditional EISA method, an evaporation-induced aggregation assembly (EIAA) mechanism, was proposed [150]. This solution precipitation method can produce mesoporous materials with various shapes including spheres, fibers, and polyhedrons in the form of powder [151]. Moreover, the EIAA method tolerates a greater portion of water compared to EISA, which makes it more suitable for scaling up. In detail, the water-insoluble template PEO-*b*-PMMA and silica precursor were first dissolved in a mixture of THF and water. As THF evaporated, the BCP and silicate oligomers were driven to form composite micelles with silicates located at the shell. With further removal of the solvent, the micelles assembled into mesostructured particles at the liquid-liquid interface and precipitated from the solution. Subsequent calcination of the precipitants completely removed the PMMA block to form pores. A schematic illustration of EIAA is shown in Figure 20. 

**Figure 21:**
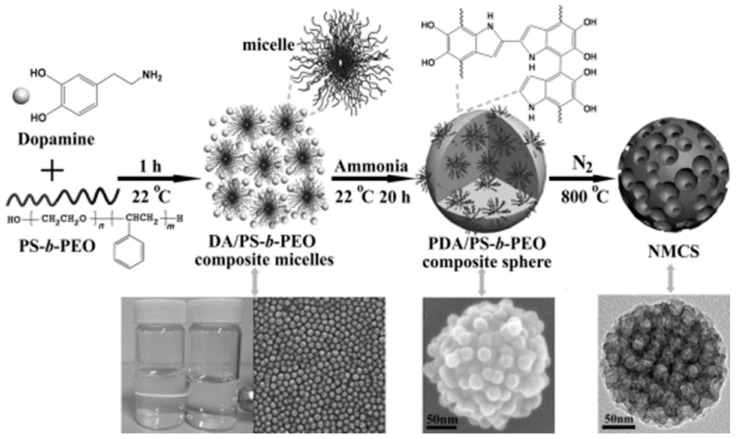
Synthesis of nitrogen-doped mesoporous carbon spheres (NMCS) with extra-large pores through the assembly of diblock copolymer micelles. Reprinted from Reference [152]. (Copyright (2015) WILEY-VCH Verlag GmbH & Co. KGaA, Weinheim).

More recently, Tang et al. proposed a facile micelle method to synthesize N-doped mesoporous carbon spheres without the EISA process (Figure 21). The key difference of this research from EISA lies in the formation of stable micelles of dopamine (both C and N sources) using a high molecular weight PS-*b*-PEO as a template. After the micelle was formed, dopamine was polymerized using ammonia as an initiator. These “frozen” micelles joined together forming into larger spheres with sizes around 200 nm. These composite spheres were easily separated from the solution through centrifugation and allowed for further carbonization to remove the template and fabricate mesoporous spheres [152]. 

Many studies demonstrated the advantage of mesopores over micropores in the mass transport process in a variety of applications [135,153]. Larger pore size facilitates a more efficient diffusion of guest molecules within the pores [136], and also allows the loading of metal catalyst particles [154,155]. Thick pore walls are also favorable because of the possibility for further modification on the pore surface [151,155]. 

Since the pores are produced from the calcination of the hydrophobic segment of the BCP, high molecular weight of the sacrificial block is highly desired. Unfortunately, the mesoporous materials templated from commercial Pluronic copolymers show a pore size limited to 12 nm and a wall thickness no more than 6 nm [151]. BCPs with higher molecular weight in the sacrificial segment (higher hydrophobic volume in the copolymer) have been extensively utilized for larger pores in the resulting porous material. Using PEO-*b*-PS (*M*_w_ = 29,700 g/mol) as the copolymer template, adding a special hydrothermal treatment step, Deng et al. achieved mesoporous silica with pore diameters as large as 30.8 nm [156]. By varying the lengths of each segment of BCP templates, Tang et al. observed a strong correlation of the resulting pore size with the length of BCP, where PS_37_-*b*-PEO_114_, PS_178_-*b*-PEO_886_, and PS_173_-*b*-PEO_170_ resulted in pore sizes of 5.4 nm, 16 nm and 16 nm, respectively [152]. In another case, aluminum-organophosphonate films with tunable macropores (30–200 nm) were synthesized via ultra-high molecular weight PEO-*b*-PS (250,000 g/mol) templates, though the resulting material exhibited a relatively disordered mesostructure [157]. In addition to utilizing high molecular weight BCPs as templates to target material with larger pores, an additional hydrophobic component can be added to the mixture as a pore swelling agent. For example, with the aid of trimethylbenzene (TMB), the pore size of silica can be expanded to 30 nm [158]. Moreover, Deng et al. reported the use of PS homopolymer as the pore expanding agent in the synthesis of mesoporous carbon, when PS-*b*-PEO is selected as the template [159]. However, care must be taken when using the pore expanding agent to the composite in order to avoid the risk of unstable mesostructures [160]. 

**Figure 22:**
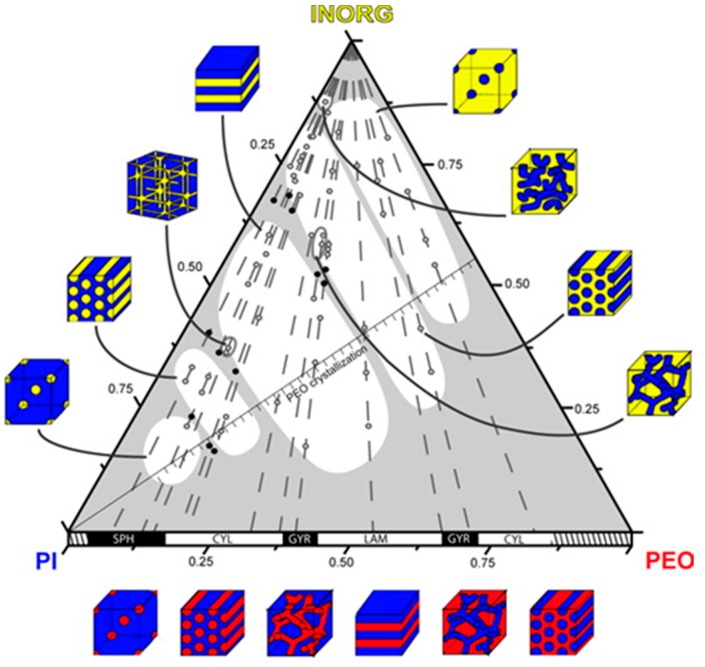
Ternary diagram mapping out the morphologies found for various composites directed by PI-*b*-PEO using (3-glycidyloxypropyl)trimethoxysilane (GLYMO) and aluminum sec-butoxide as inorganic precursors. At the bottom of the diagram, schematics of the morphologies found for the pure PI-*b*-PEO are shown. Hatched areas along the PI-*b*-PEO axis indicate areas where no data was available from the diblock copolymer diagram. Each white region within the diagram is labeled with a schematic representing the morphology of the composites formed. The yellow (light) regions in these schematic morphologies on the right and left are a representation of the PEO/inorganic domains. Closed dark points on a gray background indicate areas where biphasic behavior is observed. Reprinted from Reference [161]. (Copyright (2009) American Chemical Society).

The arrangement of a mesostructured material is closely related to the microphase separation of the BCP template. Interestingly, simply varying the amount of the precursor with respect to the copolymer would also lead to morphological changes in the composite system [148]. Garcia and coworkers fully explored the phase diagram of a polymer/alumina/silane composite system aimed for the synthesis of porous aluminosilicate using PEO-*b*-PI as a template. As illustrated in Figure 22, eight morphologies of the composite system were obtained by adding different amounts of the inorganic precursor into BCP templates [161]. Many interesting crystal symmetries could be achieved through fine tuning the template compositions, and examples are substantially reviewed [151].

## 5. Conclusions and Perspectives

In this review, we have discussed the robust synthetic approaches to well-defined block copolymers with controlled compositions and architectures, in which the sequential addition of controlled/living polymerization techniques and the combination of controlled polymerization techniques with facile coupling chemistry have expanded the spectrum of available BCPs. One of the most fascinating features of BCPs is their ability to self-assemble into nanoscale ordered structures with various morphologies such as spheres, cylinders, bicontinuous structures, lamellae, etc. Detailed study both from experiments and theoretical modeling has revealed that the nature of morphologies is determined by factors including interaction parameter (χ), degree of polymerization (N), and the volume fraction (*f*), as well as molecular architecture. Due to these unique features, BCPs have attracted massive attention as thermoplastic elastomers, as drug delivery systems, in soft lithography, as mesoporous materials, and in many other areas. 

These fascinating BCPs with chemical and topological diversity bring new challenges for the establishment of structure-property relationships and novel applications. For instance, BCP materials including rod–coil diblocks and bottlebrush materials should be investigated to create more elaborate nanostructures. Recent advancements in BCP patterning have pushed the limits of feature sizes to both smaller (<5 nm) and larger scales (>50 nm). We expect that these new challenges can be coped with by precisely tunable interactions between blocks in terms of range and strength, which further relies on their precise synthesis and guidance from theory and simulation. Strategical placement of ionic moieties might offer a promising approach. Although significant work has been reported, more interdisciplinary collaborations are still needed in order to advance the synthesis, structural and dynamic characterization, theory and simulation, and to gain a clear picture of BCP structure-property relationships.

## References

[1] Hamley I.W. (1998). The Physics of Block Copolymers.

[2] Hadjichristidis N., Iatrou H., Pitsikalis M., Mays J. (2006). Macromolecular architectures by living and controlled/living polymerizations. Prog. Polym. Sci..

[3] Uhrig D., Mays J.W. (2005). Experimental techniques in high-vacuum anionic polymerization. J. Polym. Sci. Part A Polym. Chem..

[4] Zhang H., Hong K., Mays J.W. (2002). Synthesis of block copolymers of styrene and methyl methacrylate by conventional free radical polymerization in room temperature ionic liquids. Macromolecules.

[5] Uhrig D., Mays J.W. (2002). Synthesis of combs, centipedes, and barbwires: Poly (isoprene-graft-styrene) regular multigraft copolymers with trifunctional, tetrafunctional, and hexafunctional branch points. Macromolecules.

[6] Hong K., Uhrig D., Mays J.W. (1999). Living anionic polymerization. Curr. Opin. Solid State Mater. Sci..

[7] Pochan D.J., Gido S.P., Pispas S., Mays J.W., Ryan A.J., Fairclough J.P.A., Hamley I.W., Terrill N.J. (1996). Morphologies of microphase-separated A2B simple graft copolymers. Macromolecules.

[8] Bates F.S., Fredrickson G.H. (1999). Block copolymers—Designer soft materials. Phys. Today.

[9] Lennon E.M., Katsov K., Fredrickson G.H. (2008). Free energy evaluation in field-theoretic polymer simulations. Phys. Rev. Lett..

[10] Matsen M.W., Bates F.S. (1996). Origins of complex self-assembly in block copolymers. Macromolecules.

[11] Poelma J.E., Ono K., Miyajima D., Aida T., Satoh K., Hawker C.J. (2012). Cyclic block copolymers for controlling feature sizes in block copolymer lithography. ACS Nano.

[12] Kim H.-C., Park S.-M., Hinsberg W.D. (2009). Block copolymer based nanostructures: Materials, processes, and applications to electronics. Chem. Rev..

[13] Hawker C.J., Wooley K.L. (2005). The convergence of synthetic organic and polymer chemistries. Science.

[14] Fan F., Wang W.Y., Holt A.P., Feng H.B., Uhrig D., Lu X.Y., Hong T., Wang Y.Y., Kang N.G., Mays J. (2016). Effect of molecular weight on the ion transport mechanism in polymerized ionic liquids. Macromolecules.

[15] Matyjaszewski K., Spanswick J. (2005). Controlled/living radical polymerization. Mater. Today.

[16] Matyjaszewski K., Sumerlin B.S., Tsarevsky N.V. (2012). Progress in Controlled Radical Polymerization: Mechanisms and Techniques.

[17] Braunecker W.A., Matyjaszewski K. (2007). Controlled/living radical polymerization: Features, developments, and perspectives. Prog. Polym. Sci..

[18] Gody G., Zetterlund P.B., Perrier S., Harrisson S. (2016). The limits of precision monomer placement in chain growth polymerization. Nat. Commun..

[19] Cao P.-F., Wojnarowska Z., Hong T., Carroll B., Li B., Feng H., Parsons L., Wang W., Lokitz B.S., Cheng S. (2017). A star-shaped single lithium-ion conducting copolymer by grafting a poss nanoparticle. Polymer.

[20] Wang J.-S., Matyjaszewski K. (1995). “Living”/controlled radical polymerization. Transition-metal-catalyzed atom transfer radical polymerization in the presence of a conventional radical initiator. Macromolecules.

[21] Nicolas J., Guillaneuf Y., Lefay C., Bertin D., Gigmes D., Charleux B. (2013). Nitroxide-mediated polymerization. Prog. Polym. Sci..

[22] Orilall M.C., Wiesner U. (2011). Block copolymer based composition and morphology control in nanostructured hybrid materials for energy conversion and storage: Solar cells, batteries, and fuel cells. Chem. Soc. Rev..

[23] Li C., Tang Y., Armes S.P., Morris C.J., Rose S.F., Lloyd A.W., Lewis A.L. (2005). Synthesis and characterization of biocompatible thermo-responsive gelators based on aba triblock copolymers. Biomacromolecules.

[24] Ding H.J., Park S., Zhong M.J., Pan X.C., Pietrasik J., Bettinger C.J., Matyjaszewski K. (2016). Facile arm-first synthesis of star block copolymers via arget atrp with ppm amounts of catalyst. Macromolecules.

[25] Min K., Gao H., Matyjaszewski K. (2005). Preparation of homopolymers and block copolymers in miniemulsion by atrp using activators generated by electron transfer (aget). J. Am. Chem. Soc..

[26] Min K., Gao H., Matyjaszewski K. (2007). Use of ascorbic acid as reducing agent for synthesis of well-defined polymers by arget atrp. Macromolecules.

[27] Kwak Y., Matyjaszewski K. (2009). Arget atrp of methyl methacrylate in the presence of nitrogen-based ligands as reducing agents. Polym. Int..

[28] Siegwart D.J., Oh J.K., Matyjaszewski K. (2012). Atrp in the design of functional materials for biomedical applications. Prog. Polym. Sci..

[29] Sugihara S., Sugihara K., Armes S.P., Ahmad H., Lewis A.L. (2010). Synthesis of biomimetic poly (2-(methacryloyloxy) ethyl phosphorylcholine) nanolatexes via atom transfer radical dispersion polymerization in alcohol/water mixtures. Macromolecules.

[30] Ding A., Lu G., Guo H., Huang X. (2017). Double-bond-containing polyallene-based triblock copolymers via phenoxyallene and (meth)acrylate. Sci. Rep..

[31] Chong Y., Le T.P., Moad G., Rizzardo E., Thang S.H. (1999). A more versatile route to block copolymers and other polymers of complex architecture by living radical polymerization: The raft process. Macromolecules.

[32] You Y., Hong C., Wang W., Lu W., Pan C. (2004). Preparation and characterization of thermally responsive and biodegradable block copolymer comprised of pnipaam and pla by combination of rop and raft methods. Macromolecules.

[33] Chaduc I., Zhang W., Rieger J., Lansalot M., D’Agosto F., Charleux B. (2011). Amphiphilic block copolymers from a direct and one-pot raft synthesis in water. Macromol. Rapid Commun..

[34] Wang X., Luo Y., Li B., Zhu S. (2009). Ab initio batch emulsion raft polymerization of styrene mediated by poly(acrylic acid-*b*-styrene) trithiocarbonate. Macromolecules.

[35] Luo Y., Wang X., Li B.-G., Zhu S. (2011). Toward well-controlled ab initio raft emulsion polymerization of styrene mediated by 2-(((dodecylsulfanyl)carbonothioyl)sulfanyl)propanoic acid. Macromolecules.

[36] Luo Y., Wang X., Zhu Y., Li B.-G., Zhu S. (2010). Polystyrene-block-poly (*n*-butyl acrylate)-block-polystyrene triblock copolymer thermoplastic elastomer synthesized via raft emulsion polymerization. Macromolecules.

[37] Benoit D., Harth E., Fox P., Waymouth R.M., Hawker C.J. (2000). Accurate structural control and block formation in the living polymerization of 1, 3-dienes by nitroxide-mediated procedures. Macromolecules.

[38] Szwarc M., Levy M., Milkovich R. (1956). Polymerization initiated by electron transfer to monomer. A new method of formation of block polymers1. J. Am. Chem. Soc..

[39] Szwarc M. (1956). ‘Living’ polymers. Nature.

[40] Pitsikalis M., Pispas S., Mays J.W., Hadjichristidis N. (1998). Nonlinear block copolymer architectures. Blockcopolymers-Polyelectrolytes-Biodegradation.

[41] Hirao A., Loykulnant S., Ishizone T. (2002). Recent advance in living anionic polymerization of functionalized styrene derivatives. Prog. Polym. Sci..

[42] Matsuo A., Watanabe T., Hirao A. (2004). Synthesis of well-defined dendrimer-like branched polymers and block copolymer by the iterative approach involving coupling reaction of living anionic polymer and functionalization. Macromolecules.

[43] Advincula R., Zhou Q., Park M., Wang S., Mays J., Sakellariou G., Pispas S., Hadjichristidis N. (2002). Polymer brushes by living anionic surface initiated polymerization on flat silicon (sio x) and gold surfaces: Homopolymers and block copolymers. Langmuir.

[44] Hirao A., Hayashi M., Haraguchi N. (2000). Synthesis of well-defined functionalized polymers and star branched polymers by means of living anionic polymerization using specially designed 1,1-diphenylethylene derivatives. Macromol. Rapid Commun..

[45] Zhou Q., Fan X., Xia C., Mays J., Advincula R. (2001). Living anionic surface initiated polymerization (sip) of styrene from clay surfaces. Chem. Mater..

[46] Wojnarowska Z., Feng H., Fu Y., Cheng S., Carroll B., Kumar R., Novikov V.N., Kisliuk A.M., Saito T., Kang N.-G. (2017). Effect of chain rigidity on the decoupling of ion motion from segmental relaxation in polymerized ionic liquids: Ambient and elevated pressure studies. Macromolecules.

[47] Wojnarowska Z., Feng H., Diaz M., Ortiz A., Ortiz I., Knapik-Kowalczuk J., Vilas M., Verdía P., Tojo E., Saito T. (2017). Revealing the charge transport mechanism in polymerized ionic liquids: Insight from high pressure conductivity studies. Chem. Mater..

[48] Matsuo Y., Konno R., Ishizone T., Goseki R., Hirao A. (2013). Precise synthesis of block polymers composed of three or more blocks by specially designed linking methodologies in conjunction with living anionic polymerization system. Polymers.

[49] Hadjichristidis N., Pitsikalis M., Pispas S., Iatrou H. (2001). Polymers with complex architecture by living anionic polymerization. Chem. Rev..

[50] Ekizoglou N., Hadjichristidis N. (2002). Synthesis of model linear tetrablock quaterpolymers and pentablock quintopolymers of ethylene oxide. J. Polym. Sci. Part A Polym. Chem..

[51] Bellas V., Iatrou H., Pitsinos E.N., Hadjichristidis N. (2001). Heterofunctional linking agents for the synthesis of well-defined block copolymers of dimethylsiloxane and *tert*-butyl methacrylate or 2-vinylpyridine. Macromolecules.

[52] Iatrou H., Mays J.W., Hadjichristidis N. (1998). Regular comb polystyrenes and graft polyisoprene/polystyrene copolymers with double branches (“centipedes”). Quality of (1,3-phenylene) bis (3-methyl-1-phenylpentylidene) dilithium initiator in the presence of polar additives. Macromolecules.

[53] Quirk R.P., Yoo T., Lee Y., Kim J., Lee B. (2000). Applications of 1,1-diphenylethylene chemistry in anionic synthesis of polymers with controlled structures. Biopolymers PVA Hydrogels, Anionic Polymerisation Nanocomposites.

[54] Handlin D.L., Hansen D.R., Wright K.J., Trenor S.R. (2010). Industrial applications. Controlled and Living Polymerizations: From Mechanisms to Applications.

[55] Wang H., Lu W., Wang W., Shah P.N., Misichronis K., Kang N.-G., Mays J.W. (2017). Design and synthesis of multigraft copolymer thermoplastic elastomers: Superelastomers. Macromol. Chem. Phys..

[56] Aydogan C., Kutahya C., Allushi A., Yilmaz G., Yagci Y. (2017). Block copolymer synthesis in one shot: Concurrent metal-free atrp and rop processes under sunlight. Polym. Chem..

[57] Opsteen J.A., van Hest J.C. (2005). Modular synthesis of block copolymers via cycloaddition of terminal azide and alkyne functionalized polymers. Chem. Commun. (Camb.).

[58] Yamamoto T., Tezuka Y. (2011). Topological polymer chemistry: A cyclic approach toward novel polymer properties and functions. Polym. Chem..

[59] Keddie D.J. (2014). A guide to the synthesis of block copolymers using reversible-addition fragmentation chain transfer (raft) polymerization. Chem. Soc. Rev..

[60] Inglis A.J., Sinnwell S., Davis T.P., Barner-Kowollik C., Stenzel M.H. (2008). Reversible addition fragmentation chain transfer (raft) and hetero-diels–alder chemistry as a convenient conjugation tool for access to complex macromolecular designs. Macromolecules.

[61] Lohmeijer B.G., Schubert U.S. (2002). Supramolecular engineering with macromolecules: An alternative concept for block copolymers. Angew. Chem. Int. Ed..

[62] Guo Z., Zhang G., Qiu F., Zhang H., Yang Y., Shi A.-C. (2008). Discovering ordered phases of block copolymers: New results from a generic fourier-space approach. Phys. Rev. Lett..

[63] Förster S., Plantenberg T. (2002). From self-organizing polymers to nanohybrid and biomaterials. Angew. Chem. Int. Ed..

[64] Kim J.K., Yang S.Y., Lee Y., Kim Y. (2010). Functional nanomaterials based on block copolymer self-assembly. Prog. Polym. Sci..

[65] Bates F.S. (1991). Polymer-polymer phase behavior. Science.

[66] Matsen M.W., Schick M. (1994). Stable and unstable phases of a diblock copolymer melt. Phys. Rev. Lett..

[67] Honeker C.C., Thomas E.L. (1996). Impact of morphological orientation in determining mechanical properties in triblock copolymer systems. Chem. Mater..

[68] DeRouchey J., Thurn-Albrecht T., Russell T., Kolb R. (2004). Block copolymer domain reorientation in an electric field: An in-situ small-angle x-ray scattering study. Macromolecules.

[69] Mai Y., Eisenberg A. (2012). Self-assembly of block copolymers. Chem. Soc. Rev..

[70] Storey R.F., Baugh D. (2000). Poly (styrene-b-isobutylene-b-styrene) block copolymers and ionomers therefrom: Morphology as determined by small-angle x-ray scattering and transmission electron microscopy. Polymer.

[71] Elabd Y.A., Napadensky E., Walker C.W., Winey K.I. (2006). Transport properties of sulfonated poly (styrene-*b*-isobutylene-*b*-styrene) triblock copolymers at high ion-exchange capacities. Macromolecules.

[72] Goswami M., Sumpter B.G., Huang T., Messman J.M., Gido S.P., Isaacs-Sodeye A., Mays J.W. (2010). Tunable morphologies from charged block copolymers. Soft Matter.

[73] Sanoja G.E., Popere B.C., Beckingham B.S., Evans C.M., Lynd N.A., Segalman R.A. (2016). Structure–conductivity relationships of block copolymer membranes based on hydrated protic polymerized ionic liquids: Effect of domain spacing. Macromolecules.

[74] Patel S.N., Javier A.E., Beers K.M., Pople J.A., Ho V., Segalman R.A., Balsara N.P. (2012). Morphology and thermodynamic properties of a copolymer with an electronically conducting block: Poly(3-ethylhexylthiophene)-block-poly (ethylene oxide). Nano Lett..

[75] Robbins S.W., Beaucage P.A., Sai H., Tan K.W., Werner J.G., Sethna J.P., DiSalvo F.J., Gruner S.M., Van Dover R.B., Wiesner U. (2016). Block copolymer self-assembly-directed synthesis of mesoporous gyroidal superconductors. Sci. Adv..

[76] Kim K., Schulze M.W., Arora A., Lewis R.M., Hillmyer M.A., Dorfman K.D., Bates F.S. (2017). Thermal processing of diblock copolymer melts mimics metallurgy. Science.

[77] Feng H., Changez M., Hong K., Mays J.W., Kang N.-G. (2017). 2-isopropenyl-2-oxazoline: Well-defined homopolymers and block copolymers via living anionic polymerization. Macromolecules.

[78] Hamley I.W. (2003). Nanostructure fabrication using block copolymers. Nanotechnology.

[79] Alexandridis P., Lindman B. (2000). Amphiphilic Block Copolymers: Self-Assembly and Applications.

[80] Blanazs A., Armes S.P., Ryan A.J. (2009). Self-assembled block copolymer aggregates: From micelles to vesicles and their biological applications. Macromol. Rapid Commun..

[81] Zhang L., Eisenberg A. (1996). Multiple morphologies and characteristics of “crew-cut” micelle-like aggregates of polystyrene-*b*-poly (acrylic acid) diblock copolymers in aqueous solutions. J. Am. Chem. Soc..

[82] Wang X., Guerin G., Wang H., Wang Y., Manners I., Winnik M.A. (2007). Cylindrical block copolymer micelles and co-micelles of controlled length and architecture. Science.

[83] Honda S., Yamamoto T., Tezuka Y. (2010). Topology-directed control on thermal stability: Micelles formed from linear and cyclized amphiphilic block copolymers. J. Am. Chem. Soc..

[84] Jain S., Bates F.S. (2003). On the origins of morphological complexity in block copolymer surfactants. Science.

[85] Wang W. (2015). Novel Thermoplastic Elastomers Based on Benzofulvene: Synthesis and Mechanical Properties. Ph.D. Thesis.

[86] Bhowmick A.K., Stephens H. (2000). Handbook of Elastomers.

[87] Hadjichristidis N., Pispas S., Floudas G. (2003). Block Copolymers: Synthetic Strategies, Physical Properties, and Applications.

[88] Xenidou M., Hadjichristidis N. (1998). Synthesis of model multigraft copolymers of butadiene with randomly placed single and double polystyrene branches. Macromolecules.

[89] Fetters L.J., Morton M. (1969). Synthesis and properties of block polymers. I. Poly-α-methylstyrene-polyisoprene-poly-α-methylstyrene. Macromolecules.

[90] Bolton J.M., Hillmyer M.A., Hoye T.R. (2014). Sustainable thermoplastic elastomers from terpene-derived monomers. ACS Macro Lett..

[91] Fetters L., Firer E., Dafauti M. (1977). Synthesis and properties of block copolymers. 4. Poly(*p*-*tert*-butylstyrene-diene-*p*-*tert*-butylstyrene) and poly (*p*-*tert*-butylstyrene-isoprene-styrene). Macromolecules.

[92] Wang W.Y., Schlegel R., White B.T., Williams K., Voyloy D., Steren C.A., Goodwin A., Coughlin E.B., Gido S., Beiner M. (2016). High temperature thermoplastic elastomers synthesized by living anionic polymerization in hydrocarbon solvent at room temperatuie. Macromolecules.

[93] Lu W., Wang Y., Wang W., Cheng S., Zhu J., Xu Y., Hong K., Kang N.-G., Mays J. (2017). All acrylic-based thermoplastic elastomers with high upper service temperature and superior mechanical properties. Polym. Chem..

[94] Martín-Gomis L., Fernández-García M., de la Fuente J.L., Madruga E.L., Cerrada M.L. (2003). Physical properties of PBMA-*b*-PBA-*b*-PBMA triblock copolymers synthesized by atom transfer radical polymerization. Macromol. Chem. Phys..

[95] Tong J., Leclère P., Doneux C., Brédas J.-L., Lazzaroni R., Jérôme R. (2001). Morphology and mechanical properties of poly (methylmethacrylate)-*b*-poly (alkylacrylate)-*b*-poly (methylmethacrylate). Polymer.

[96] Zhu Y.Q., Burgaz E., Gido S.P., Staudinger U., Weidisch R., Uhrig D., Mays J.W. (2006). Morphology and tensile properties of multigraft copolymers with regularly spaced tri-, tetra-, and hexafunctional junction points. Macromolecules.

[97] Staudinger U., Weidisch R., Zhu Y., Gido S., Uhrig D., Mays J., Iatrou H., Hadjichristidis N. (2006). Mechanical properties and hysteresis behaviour of multigraft copolymers. Macromol. Symp..

[98] Wang W., Wang W., Lu X., Bobade S., Chen J., Kang N.-G., Zhang Q., Mays J. (2014). Synthesis and characterization of comb and centipede multigraft copolymers PnBA-*g*-PS with high molecular weight using miniemulsion polymerization. Macromolecules.

[99] Wang W., Wang W., Li H., Lu X., Chen J., Kang N.-G., Zhang Q., Mays J. (2015). Synthesis and characterization of graft copolymers poly (isoprene-*g*-styrene) of high molecular weight by a combination of anionic polymerization and emulsion polymerization. Ind. Eng. Chem. Res..

[100] Rösler A., Vandermeulen G.W., Klok H.-A. (2012). Advanced drug delivery devices via self-assembly of amphiphilic block copolymers. Adv. Drug Deliv. Rev..

[101] O’Reilly R.K., Hawker C.J., Wooley K.L. (2006). Cross-linked block copolymer micelles: Functional nanostructures of great potential and versatility. Chem. Soc. Rev..

[102] Wei H., Cheng S.-X., Zhang X.-Z., Zhuo R.-X. (2009). Thermo-sensitive polymeric micelles based on poly (n-isopropylacrylamide) as drug carriers. Prog. Polym. Sci..

[103] Lee H.-N., Bai Z., Newell N., Lodge T.P. (2010). Micelle/inverse micelle self-assembly of a peo-pnipam block copolymer in ionic liquids with double thermoresponsivity. Macromolecules.

[104] Roy D., Brooks W.L., Sumerlin B.S. (2013). New directions in thermoresponsive polymers. Chem. Soc. Rev..

[105] Thambi T., Son S., Lee D.S., Park J.H. (2016). Poly(ethylene glycol)-*b*-poly (lysine) copolymer bearing nitroaromatics for hypoxia-sensitive drug delivery. Acta Biomater..

[106] Ahmed F., Discher D.E. (2004). Self-porating polymersomes of peg–pla and peg–pcl: Hydrolysis-triggered controlled release vesicles. J. Control. Release.

[107] Zupancich J.A., Bates F.S., Hillmyer M.A. (2006). Aqueous dispersions of poly (ethylene oxide)-*b*-poly (γ-methyl-ε-caprolactone) block copolymers. Macromolecules.

[108] Meng F., Engbers G.H., Feijen J. (2005). Biodegradable polymersomes as a basis for artificial cells: Encapsulation, release and targeting. J. Control. Release.

[109] Ghoroghchian P.P., Li G., Levine D.H., Davis K.P., Bates F.S., Hammer D.A., Therien M.J. (2006). Bioresorbable vesicles formed through spontaneous self-assembly of amphiphilic poly(ethylene oxide)-block-polycaprolactone. Macromolecules.

[110] Discher B.M., Bermudez H., Hammer D.A., Discher D.E., Won Y.-Y., Bates F.S. (2002). Cross-linked polymersome membranes: Vesicles with broadly adjustable properties. J. Phys. Chem. B.

[111] Pang Z., Lu W., Gao H., Hu K., Chen J., Zhang C., Gao X., Jiang X., Zhu C. (2008). Preparation and brain delivery property of biodegradable polymersomes conjugated with ox26. J. Control. Release.

[112] Bates C.M., Maher M.J., Janes D.W., Ellison C.J., Willson C.G. (2013). Block copolymer lithography. Macromolecules.

[113] Li W., Muller M. (2015). Defects in the self-assembly of block copolymers and their relevance for directed self-assembly. Annu. Rev. Chem Biomol..

[114] Seshimo T., Maeda R., Odashima R., Takenaka Y., Kawana D., Ohmori K., Hayakawa T. (2016). Perpendicularly oriented sub-10-nm block copolymer lamellae by atmospheric thermal annealing for one minute. Sci. Rep..

[115] Maher M.J., Rettner C.T., Bates C.M., Blachut G., Carlson M.C., Durand W.J., Ellison C.J., Sanders D.P., Cheng J.Y., Willson C.G. (2015). Directed self-assembly of silicon-containing block copolymer thin films. ACS Appl. Mater. Interfaces.

[116] Schulze M.W., McIntosh L.D., Hillmyer M.A., Lodge T.P. (2013). High-modulus, high-conductivity nanostructured polymer electrolyte membranes via polymerization-induced phase separation. Nano Lett..

[117] Kennemur J.G., Hillmyer M.A., Bates F.S. (2012). Synthesis, thermodynamics, and dynamics of poly (4-*tert*-butylstyrene-*b*-methyl methacrylate). Macromolecules.

[118] Keen I., Yu A., Cheng H.-H., Jack K.S., Nicholson T.M., Whittaker A.K., Blakey I. (2012). Control of the orientation of symmetric poly (styrene)-block-poly (d,l-lactide) block copolymers using statistical copolymers of dissimilar composition. Langmuir.

[119] Ham S., Shin C., Kim E., Ryu D.Y., Jeong U., Russell T.P., Hawker C.J. (2008). Microdomain orientation of ps-*b*-pmma by controlled interfacial interactions. Macromolecules.

[120] Mansky P., Liu Y., Huang E., Russell T., Hawker C. (1997). Controlling polymer-surface interactions with random copolymer brushes. Science.

[121] Mansky P., Russell T., Hawker C., Mays J., Cook D., Satija S. (1997). Interfacial segregation in disordered block copolymers: Effect of tunable surface potentials. Phys. Rev. Lett..

[122] Zhao Y., Sivaniah E., Hashimoto T. (2008). Saxs analysis of the order-disorder transition and the interaction parameter of polystyrene-block-poly (methyl methacrylate). Macromolecules.

[123] Park S., Lee D.H., Xu J., Kim B., Hong S.W., Jeong U., Xu T., Russell T.P. (2009). Macroscopic 10-terabit–per–square-inch arrays from block copolymers with lateral order. Science.

[124] Rodwogin M.D., Spanjers C.S., Leighton C., Hillmyer M.A. (2010). Polylactide-poly(dimethylsiloxane)-polylactide triblock copolymers as multifunctional materials for nanolithographic applications. ACS Nano.

[125] Almdal K., Hillmyer M.A., Bates F.S. (2002). Influence of conformational asymmetry on polymer-polymer interactions: An entropic or enthalpic effect?. Macromolecules.

[126] Yoshida H., Suh H.S., Ramirez-Herunandez A., Lee J.I., Aida K., Wan L., Ishida Y., Tada Y., Ruiz R., de Pablo J. (2013). Topcoat approaches for directed self-assembly of strongly segregating block copolymer thin films. J. Photopolym. Sci. Technol..

[127] Cushen J.D., Otsuka I., Bates C.M., Halila S., Fort S., Rochas C., Easley J.A., Rausch E.L., Thio A., Borsali R. (2012). Oligosaccharide/silicon-containing block copolymers with 5 nm features for lithographic applications. ACS Nano.

[128] Bates C.M., Seshimo T., Maher M.J., Durand W.J., Cushen J.D., Dean L.M., Blachut G., Ellison C.J., Willson C.G. (2012). Polarity-switching top coats enable orientation of sub-10-nm block copolymer domains. Science.

[129] Ma T.-Y., Liu L., Yuan Z.-Y. (2013). Direct synthesis of ordered mesoporous carbons. Chem. Soc. Rev..

[130] Liu J., Wickramaratne N.P., Qiao S.Z., Jaroniec M. (2015). Molecular-based design and emerging applications of nanoporous carbon spheres. Nat. Mater..

[131] Wan Y., Zhao D. (2007). On the controllable soft-templating approach to mesoporous silicates. Chem. Rev..

[132] Zhai Y., Dou Y., Zhao D., Fulvio P.F., Mayes R.T., Dai S. (2011). Carbon materials for chemical capacitive energy storage. Adv. Mater..

[133] Nishihara H., Kyotani T. (2012). Templated nanocarbons for energy storage. Adv. Mater..

[134] Vu A., Qian Y., Stein A. (2012). Porous electrode materials for lithium-ion batteries–how to prepare them and what makes them special. Adv. Energy Mater..

[135] Tang J., Liu J., Torad N.L., Kimura T., Yamauchi Y. (2014). Tailored design of functional nanoporous carbon materials toward fuel cell applications. Nano Today.

[136] Rolison D.R. (2003). Catalytic nanoarchitectures—The importance of nothing and the unimportance of periodicity. Science.

[137] Slowing I.I., Trewyn B.G., Giri S., Lin V.Y. (2007). Mesoporous silica nanoparticles for drug delivery and biosensing applications. Adv. Funct. Mater..

[138] Patiño J., Gutiérrez M., Carriazo D., Ania C., Fierro J., Ferrer M., Del Monte F. (2014). Des assisted synthesis of hierarchical nitrogen-doped carbon molecular sieves for selective CO_2_ versus N_2_ adsorption. J. Mater. Chem. A.

[139] Feng H.B., Hong T., Mahurin S.M., Vogiatzis K.D., Gmernicki K.R., Long B.K., Mays J.W., Sokolov A.P., Kang N.G., Saito T. (2017). Gas separation mechanism of co2 selective amidoxime-poly(1-trimethylsilyl-1-propyne) membranes. Polym. Chem..

[140] Kamegawa T., Ishiguro Y., Seto H., Yamashita H. (2015). Enhanced photocatalytic properties of tio 2-loaded porous silica with hierarchical macroporous and mesoporous architectures in water purification. J. Mater. Chem. A.

[141] Cao Y., Huang J., Peng X., Cao D., Galaska A., Qiu S., Liu J., Khan M.A., Young D.P., Ryu J.E. (2017). Poly(vinylidene fluoride) derived fluorine-doped magnetic carbon nanoadsorbents for enhanced chromium removal. Carbon.

[142] Zhang J., Deng Y., Wei J., Sun Z., Gu D., Bongard H., Liu C., Wu H., Tu B., Schüth F. (2009). Design of amphiphilic abc triblock copolymer for templating synthesis of large-pore ordered mesoporous carbons with tunable pore wall thickness. Chem. Mater..

[143] Deng Y., Liu C., Gu D., Yu T., Tu B., Zhao D. (2008). Thick wall mesoporous carbons with a large pore structure templated from a weakly hydrophobic PEO-PMMA diblock copolymer. J. Mater. Chem..

[144] Liang C., Hong K., Guiochon G.A., Mays J.W., Dai S. (2004). Synthesis of a large-scale highly ordered porous carbon film by self-assembly of block copolymers. Angew. Chem. Int. Ed..

[145] Wei J., Sun Z., Luo W., Li Y., Elzatahry A.A., Al-Enizi A.M., Deng Y., Zhao D. (2017). New insight into the synthesis of large-pore ordered mesoporous materials. J. Am. Chem. Soc..

[146] Liang C., Li Z., Dai S. (2008). Mesoporous carbon materials: Synthesis and modification. Angew. Chem. Int. Ed..

[147] Yang P., Zhao D., Margolese D.I., Chmelka B.F., Stucky G.D. (1998). Generalized syntheses of large-pore mesoporous metal oxides with semicrystalline frameworks. Nature.

[148] Templin M., Franck A., Du Chesne A., Leist H., Zhang Y., Ulrich R., Schädler V., Wiesner U. (1997). Organically modified aluminosilicate mesostructures from block copolymer phases. Science.

[149] Wei J., Zhou D., Sun Z., Deng Y., Xia Y., Zhao D. (2013). A controllable synthesis of rich nitrogen-doped ordered mesoporous carbon for CO_2_ capture and supercapacitors. Adv. Funct. Mater..

[150] Wei J., Wang H., Deng Y., Sun Z., Shi L., Tu B., Luqman M., Zhao D. (2011). Solvent evaporation induced aggregating assembly approach to three-dimensional ordered mesoporous silica with ultralarge accessible mesopores. J. Am. Chem. Soc..

[151] Deng Y., Wei J., Sun Z., Zhao D. (2013). Large-pore ordered mesoporous materials templated from non-pluronic amphiphilic block copolymers. Chem. Soc. Rev..

[152] Tang J., Liu J., Li C., Li Y., Tade M.O., Dai S., Yamauchi Y. (2015). Synthesis of nitrogen-doped mesoporous carbon spheres with extra-large pores through assembly of diblock copolymer micelles. Angew. Chem. Int. Ed..

[153] Du H., Gan L., Li B., Wu P., Qiu Y., Kang F., Fu R., Zeng Y. (2007). Influences of mesopore size on oxygen reduction reaction catalysis of pt/carbon aerogels. J. Phys. Chem. C.

[154] Ma G., Yan X., Li Y., Xiao L., Huang Z., Lu Y., Fan J. (2010). Ordered nanoporous silica with periodic 30–60 nm pores as an effective support for gold nanoparticle catalysts with enhanced lifetime. J. Am. Chem. Soc..

[155] Deng Y., Cai Y., Sun Z., Gu D., Wei J., Li W., Guo X., Yang J., Zhao D. (2010). Controlled synthesis and functionalization of ordered large-pore mesoporous carbons. Adv. Funct. Mater..

[156] Deng Y., Yu T., Wan Y., Shi Y., Meng Y., Gu D., Zhang L., Huang Y., Liu C., Wu X. (2007). Ordered mesoporous silicas and carbons with large accessible pores templated from amphiphilic diblock copolymer poly (ethylene oxide)-*b*-polystyrene. J. Am. Chem. Soc..

[157] Kimura T. (2011). Colloidal templating fabrication of aluminum-organophosphonate films using high molecular weight PS-*b*-PEO. Chem. Asian J..

[158] Kruk M., Jaroniec M., Ko C.H., Ryoo R. (2000). Characterization of the porous structure of sba-15. Chem. Mater..

[159] Deng Y., Liu J., Liu C., Gu D., Sun Z., Wei J., Zhang J., Zhang L., Tu B., Zhao D. (2008). Ultra-large-pore mesoporous carbons templated from poly (ethylene oxide)-*b*-polystyrene diblock copolymer by adding polystyrene homopolymer as a pore expander. Chem. Mater..

[160] Yu K., Hurd A.J., Eisenberg A., Brinker C.J. (2001). Syntheses of silica/polystyrene-block-poly (ethylene oxide) films with regular and reverse mesostructures of large characteristic length scales by solvent evaporation-induced self-assembly. Langmuir.

[161] Garcia B.C., Kamperman M., Ulrich R., Jain A., Gruner S.M., Wiesner U. (2009). Morphology diagram of a diblock copolymer–aluminosilicate nanoparticle system. Chem. Mater..

